# Feasibility and resolution limits of opto-magnetic imaging of neural network activity in brain slices using color centers in diamond

**DOI:** 10.1038/s41598-018-22793-w

**Published:** 2018-03-14

**Authors:** Mürsel Karadas, Adam M. Wojciechowski, Alexander Huck, Nils Ole Dalby, Ulrik Lund Andersen, Axel Thielscher

**Affiliations:** 1 Department of Electrical Engineering, Technical University of Denmark, 2800 Kongens Lyngby, Denmark; 2 Department of Physics, Technical University of Denmark, 2800 Kongens Lyngby, Denmark; 3 Department of Drug Design and Pharmacology, Copenhagen University, 2100 Copenhagen, Denmark; 4 Danish Research Centre for Magnetic Resonance, Centre for Functional and Diagnostic Imaging and Research, Copenhagen University Hospital Hvidovre, 2650 Hvidovre, Denmark; 5 Institute of Physics, Jagiellonian University, 30-348 Kraków, Poland

## Abstract

We suggest a novel approach for wide-field imaging of the neural network dynamics of brain slices that uses highly sensitivity magnetometry based on nitrogen-vacancy (NV) centers in diamond. *In-vitro* recordings in brain slices is a proven method for the characterization of electrical neural activity and has strongly contributed to our understanding of the mechanisms that govern neural information processing. However, this traditional approach only acquires signals from a few positions, which severely limits its ability to characterize the dynamics of the underlying neural networks. We suggest to extend its scope using NV magnetometry-based imaging of the neural magnetic fields across the slice. Employing comprehensive computational simulations and theoretical analyses, we determine the spatiotemporal characteristics of the neural fields and the required key performance parameters of an NV magnetometry-based imaging setup. We investigate how the technical parameters determine the achievable spatial resolution for an optimal 2D reconstruction of neural currents from the measured field distributions. Finally, we compare the imaging of neural slice activity with that of a single planar pyramidal cell. Our results suggest that imaging of slice activity will be possible with the upcoming generation of NV magnetic field sensors, while single-shot imaging of planar cell activity remains challenging.

## Introduction

**Figure 1:**
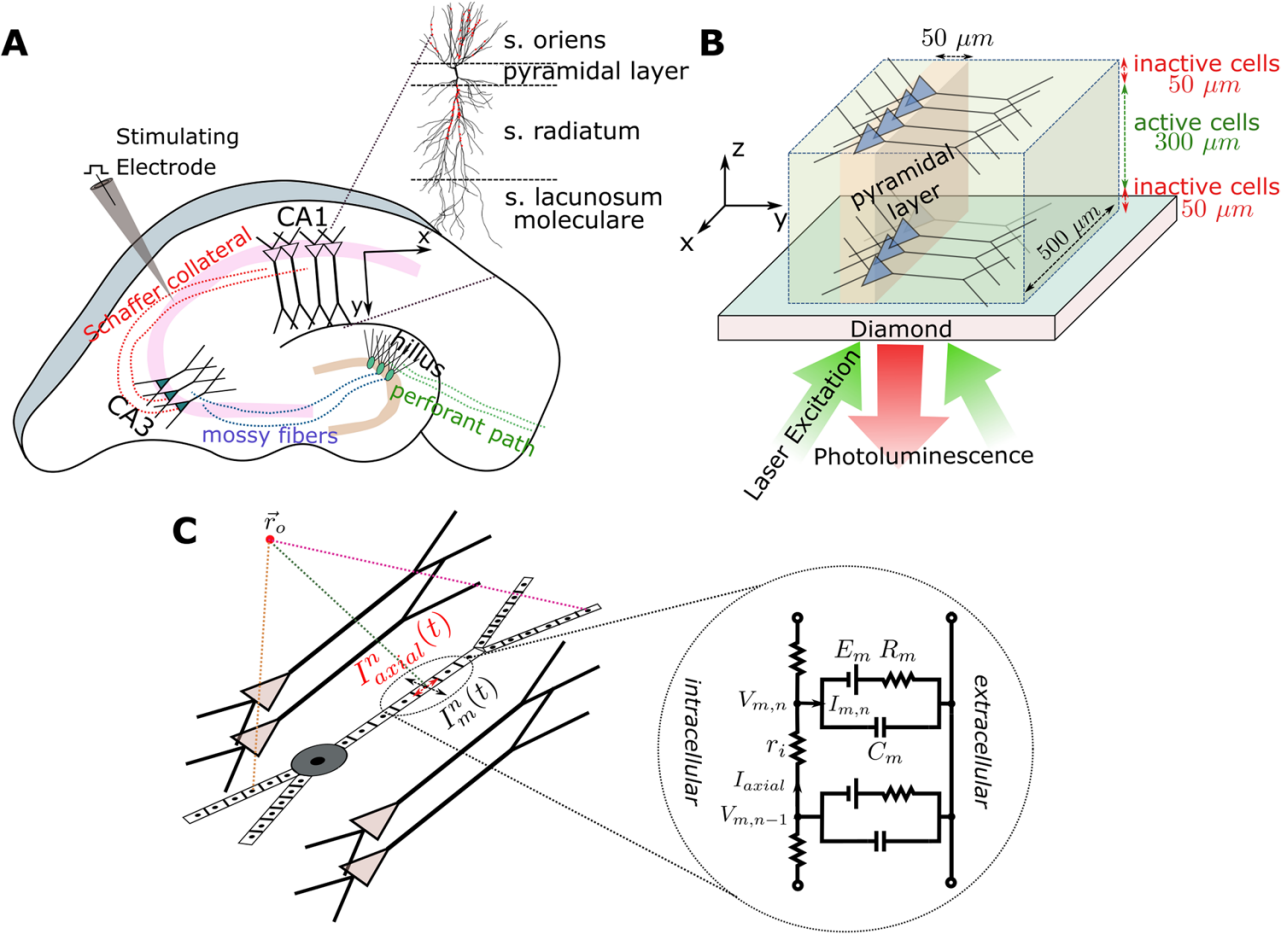
(**A**) Illustration of a hippocampus slice with its trisynaptic path. We consider the recording of neural activity from the CA1 area that is evoked by the electrical stimulation of the Schaffer collaterals. (**B)** Schematic illustration of the simulated CA1 subarea with a size of 500 × 500 × 300 µm^3^ placed on the diamond sample. It is assumed that the neural cells in a distance of up to 50 µm to the diamond are dysfunctional due to the preparation. The pyramidal cells are equally distributed in the patch along the X and Z directions, and their soma locations are randomly jittered in a 50 µm wide band in Y direction. The changes in photoluminescence emitted by the NV-layers in the diamond and caused by the neural magnetic fields can be recorded using an arrangement similar to the inverted microscope used by Barry and colleagues^10^, with the camera replacing the photodetector. (**C)** Schematic illustration of the multi-compartment model of a pyramidal cell, as applied in our forward modeling scheme.

Nitrogen-vacancy (NV) color centers in diamond are currently emerging as a practical quantum sensor to measure magnetic fields at ambient temperatures with very high sensitivity and unprecedented spatio-temporal resolution^1–3^. The approach is based on optically detected magnetic resonance^4^, where the signal is obtained through the detection of the NV fluorescence level induced by an external magnetic field. This technique opens up a wealth of new avenues for the recording of weak magnetic fields occurring in various systems such as ferromagnetic structures^5^, geological samples^6^ and electronic circuits^7^. As the quantum sensor operates under physiological conditions (room temperature and atmospheric pressure), it also provides a highly promising route for the high-resolution magnetic imaging of living biological systems^8,9^, and it might be particularly powerful in characterizing neural activity^10^. When combined with wide-field imaging sensors to read out the florescence at high spatial and temporal resolution, the approach offers the possibility to image the dynamics of neural networks in great detail (Fig. 1A,B).

*In-vitro* recordings in thin brain slices have been a mainstay in the repertoire of electrophysiological methods for the recording of neural activity. Since it was demonstrated that similar activity could be recorded in brain slices as in intact animals^11^, this approach has been used as a test bed to decipher the fundamental mechanisms that govern neural interactions. Using specialized cutting techniques, intact neural networks can be maintained and studied, while at the same time yielding good accessibility to specific neurons and neural pathways. However, the traditional method based on electrophysiology is severely limited in spatial resolution by the low number of electrodes that are used for simultaneous recordings. While detailed information is gained from a few positions on the slice, an overview of the complete network dynamics is lacking.

This has triggered new developments such as multi-electrode arrays (MEA)^12^ or voltage-sensitive dye imaging (VSDI)^13,14^ to monitor the propagation of depolarizing potentials through anatomically well-defined networks. However, as a principal limitation, it remains challenging to achieve high enough spatiotemporal resolution using existing techniques^15^. In this article, we suggest a novel, alternative method to extend the scope of slice recordings by exploiting NV-based diamond quantum sensing for the spatially resolved wide-field imaging of the neural magnetic fields. Placing the brain slice atop a two-dimensional array of NV center (of an area of 1 × 1 mm² or larger) and simultaneously performing high-resolution recordings of the fluorescence with a camera will provide detailed access to the neural dynamics across extended local networks, as for example occurring in the hippocampus region of rat or mice brains^16^ (Fig. 1A). Our analysis is based on detailed computer simulations and demonstrates the feasibility of this approach. We focus on a scenario in which magnetic fields are generated by synchronous neural activity in a subpart of the hippocampal CA1 region in a mouse brain slice, elicited by electrical stimulation of the Schaffer collaterals projecting from CA3 to CA1^17^ (Fig. 1A,B). We chose this scenario because (i) there is a comprehensive collection of data related to CA1 neurons and their models are validated by numerous experiments^18^, (ii) the topology of CA1 is well known^17^, (iii) it is easy to obtain extracellular electric/magnetic field recordings and intracellular recordings due to the regular formation of the cells, (iv) the Schaffer collateral axons form a homogeneous pathway that is easily activated to study synaptic transmission and plasticity^19^, and (v) it is simple to keep the cells in this region alive and functional during the slice preparation. The dynamic response of the CA1 pyramidal cells to the stimulation are modelled in NEURON^20^ based on multi-compartment representations of the neurons (Fig. 1C), using previously reported morphology and biophysical properties^21^. We then determine the extracellular magnetic fields at the diamond sensor surface and for comparison also the electric potential. These quantities are obtained from the simulated membrane potentials and transmembrane currents using a forward modelling scheme (see Methods and Supplementary Material). Based on comprehensive simulation results, we conclude on the expected magnetic field strengths created by the evoked neural activity and the required temporal resolution of the recording system. Finally, we simulate the optimal 2D reconstruction of the spatial distribution of the neural currents using a Wiener deconvolution filter approach. This allows us to systematically determine the achievable spatial resolution in dependence on two key technical parameters of the system, the in-plane resolution of the sensor and its noise level.

## Results

### Planar CA1 Cell

**Figure 2:**
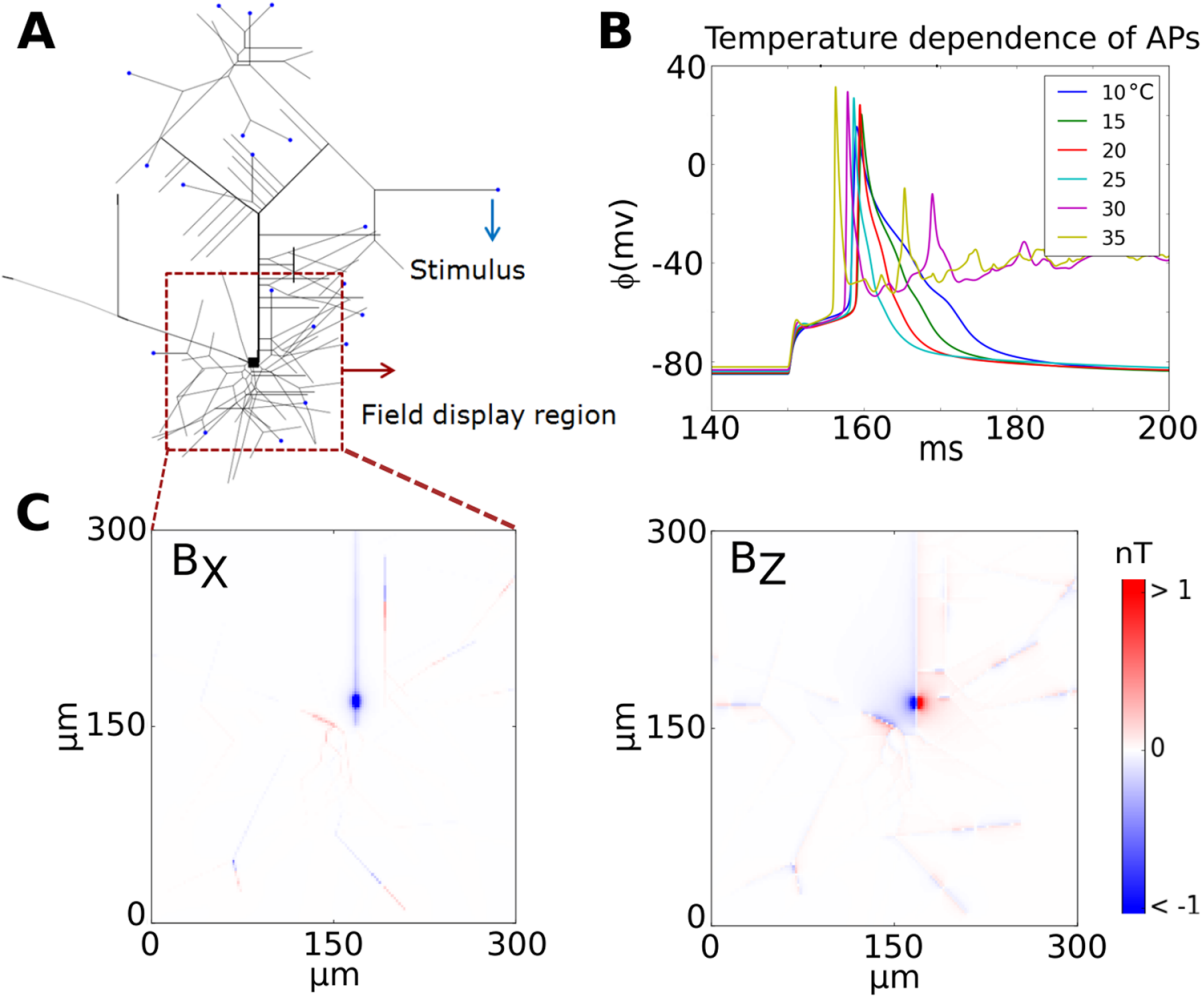
(**A)** Morphology of a single planar CA1 pyramidal cell and the current injection sites (blue dots). The cell model has 265 anatomical sections and 15 different voltage active channels. (**B)** Transmembrane potentials generated at the soma as a function of time for different temperatures. The duration of the action potential increases as the temperature decreases. (**C)** Zoomed plots of the peak B_X_ and B_Z_ components of the magnetic field close to the soma. The B_Y_ component is negligibly small. The simulated fields are assessed at a distance of 100 nm to the surface of each branch and determined at an ambient temperature of 25 °C.

We begin our analysis by re-evaluating the magnetic fields a single planar CA1 pyramidal cell^22–24^ (Fig. 2A). To simulate identical cases as tested in a prior study^24^, the neuron is stimulated with a 10 ms long injected current of 2 nA at 21 distal dendrites. The resulting transmembrane potential at the soma is plotted in Fig. 2B for different ambient temperatures. As reported^25^ the duration of the action potential increases with decreasing temperature, but only for temperatures much below ~20 °C similar durations are obtained that resemble the previously reported ones^24^. The resulting peak magnetic field distribution is shown in Fig. 2C, obtained for an ambient temperature of 25 °C. Compared to the original results^24^, our simulations suggest nearly 4–5 times smaller peak magnetic field strengths, which has several possible reasons: First, the assumed distance of 100 nm between sensor surface and the cell should be measured relative to the lower surface of the cell structure as the cell lies on the surface. In fact, we obtain similar values for the field strengths as originally reported^24^, when we measure the distance relative to the center of the cell structure. This is physically not possible, as then parts of the cells would be inside the sensor. Second, in contrast to our forward modeling scheme, the scheme used in the prior study fits the time course of the transmembrane potential by a sum of Gaussian functions. This can result in fitting errors that may contribute to the observed discrepancies. Third, the value of the ambient temperature was not mentioned in the prior study, but has a strong influence on the speed at which action potentials develop^25^. In that study, the time courses reported for the transmembrane potential are very slow and could not accurately be replicated in our simulations even when assuming an ambient temperature of 10 °C. However, neither the second nor the third reason can fully explain the differences in the reported magnetic field strengths.

### Hippocampus Slice: Spatiotemporal distribution of the neural magnetic activity

In the main scenario, we assess the stimulation of a CA1 subarea with an assumed active size of 500 × 500 × 300 µm (width × length × height) placed centrally on a diamond sensor (Fig. 1B). In order to account for dysfunctional cells caused by the preparation procedure, we add 50 µm thick subareas each on top and below the active CA1 volume, yielding a total slice thickness of 400 µm. A temperature of 35 °C is chosen. These values are within the standard thickness and temperature ranges for hippocampus slices used in electrophysiological experiments.

The magnetic field and the electric potential caused by the neural activity is calculated at the diamond surface with a size of 1 × 1 mm². Unless indicated differently, the surface is discretized using a 20 × 20 grid, resulting in an in-plane resolution of 50 µm. We simulated the electrical stimulation of the Schaffer collaterals using a temporally synchronous excitation of the excitatory AMPA (glutamatergic) synapses of the pyramidal cells, which are situated on the apical dendrites (the stratum radiatum, S.R.; Fig. 1A) and the basal dendrites (the stratum oriens, S.O.)^26^. The synaptic events occurred at two successive time points (set to 12.5 ms and 37.5 ms) to mimic a repeated electric stimulation at 40 Hz. Additionally, we included temporal jittering in our model using a normal distribution with standard deviation *σ*(σ = 1.56 ms, unless indicated differently), which mimics the jitter in the excitatory inputs that might occur due to slightly different path delays. We also varied the synaptic strength in order to distinguish between a “non-spiking” and a “spiking” case. In the non-spiking case, a strength of 0.3 nS was empirically selected that was just low enough to avoid action potentials in order to mimic strong sub-threshold activity. In the spiking case, a strength of 0.6 nS was chosen that was just high enough to reliably induce action potentials in all model neurons. Further details of the neural dynamics are stated in the Supplementary Material (chapter S5).

**Figure 3:**
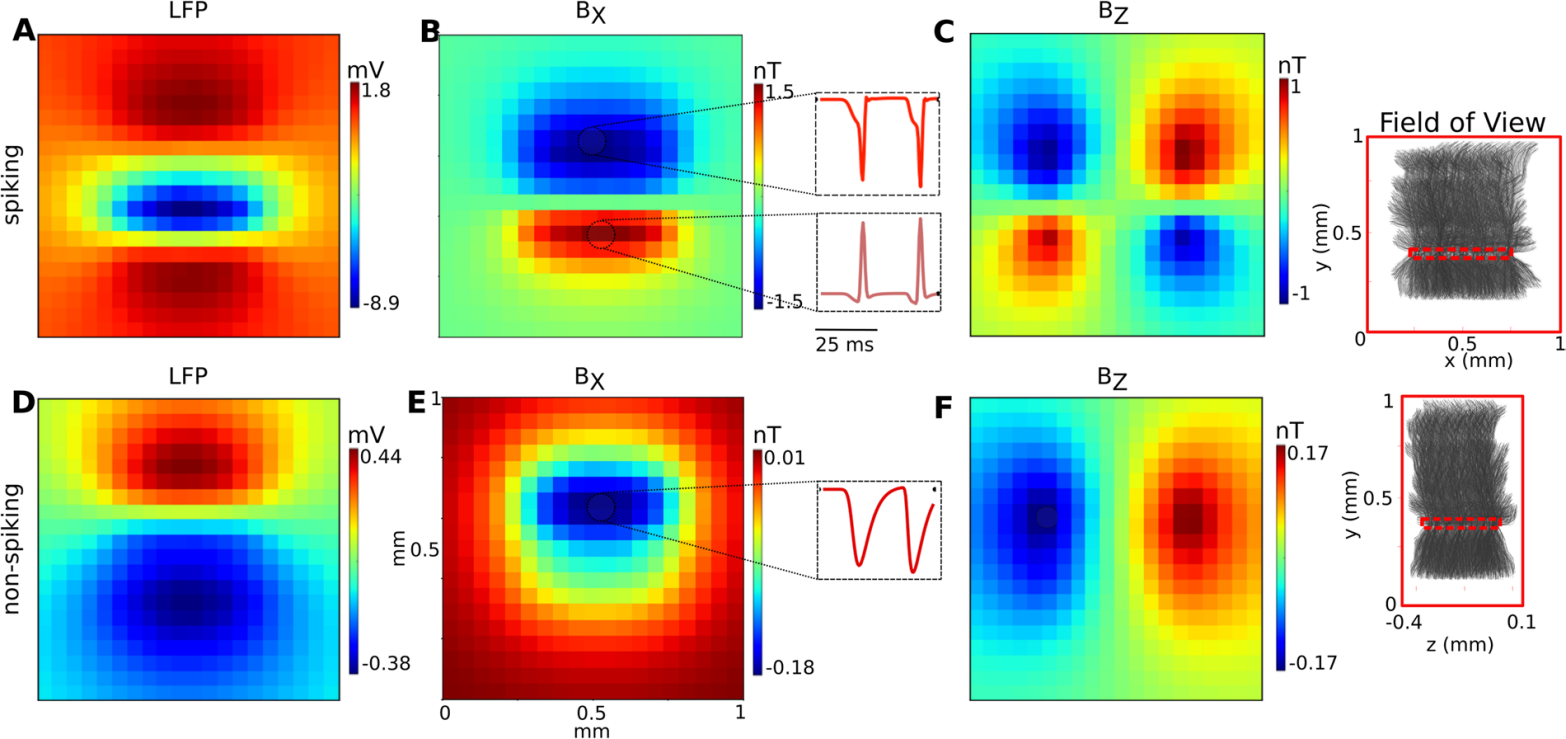
Simulation results showing the spatial distribution and the temporal shape of the extracellular fields for the CA1 subarea for the spiking case (top row, **A–C**) and the non-spiking case (bottom row, **D–F**). The insets on the right depict the size, positions and orientation of the pyramidal cells in relation to the imaged field of view. The spatial distributions are extracted at the time point when the field peaks. (**A**) The LFP for the spiking case has a large negative peak close to the soma layer, as the generation of the action potentials causes a strong current inflow. (**B**) B_X_ component of the magnetic field. The generation of the action potentials creates separate strong axial current flows in opposite directions from the S.O. and S.R. dendrites to the soma of the pyramidal cells, which leads to a negative peak in S.R. and positive peak in S.O. regions. (**C**) B_Z_ component of the magnetic field in accordance to the two opposite axial current flows from the S.O. and S.R. dendrites to the soma regions. The insets in the center depict the temporal shapes of the magnetic field components, extracted from the indicated positions above and below the pyramidal layer (a time window of 50 ms is shown). The initial phases before the action potentials reflect the accumulation of excitatory postsynaptic potentials (EPSPs). (**D**) The LFP for the non-spiking case has a dipolar distribution, with the negative peak caused by the EPSPs in the S.O. region and the positive peak above S.R. region caused by outward membrane currents which balance the excitatory synaptic currents. (**E**) B_X_ component of the magnetic field. It has a large peak around the soma, indicating an axial current flow from the S.R. to the S.O. region. (**F**) B_Z_ component of the magnetic field in accordance to the axial current flow from S.R. to S.O. The temporal shapes depicted in the insets reflect the accumulation of EPSPs.

The spatial distribution of the local field potentials (LFP) and the peak magnetic field strength for stimulation of the CA1 subarea are shown in Fig. 3. For both the spiking (Fig. 3A) and the non-spiking case (Fig. 3D), the LFPs show the expected spatial patterns and their strength is in accordance with previous studies^27–29^. The B_Y_ component of the magnetic field is negligibly small in both cases (data not shown), in accordance with the orientation of the pyramidal cells determining the neural source currents to be mainly in the Y direction. For the spiking case, the magnitude of the magnetic field B_X_ component reaches peak values of up to 1.5 nT in the sensor plane at positions directly beneath the stimulated subarea (Fig. 3B). The B_Z_ component reaches its peak value of around 1 nT at the left and right hand side of the subarea (Fig. 3C) and the spatial patterns of both the B_X_ and B_Z_ components are in accordance with source currents that mainly flow along the Y direction. In the non-spiking case (Fig. 3E,F), the peak strengths of the B_X_ component is reduced to around 0.18 nT, and the peak strength of the B_Z_ component is around 0.17 nT.

We also simulated the spatiotemporal field distributions that would occur when stimulation of the Schaffer collateral only activated the S.R. region of the pyramidal cells (Fig. S13). The peak values of the LFP and the magnetic field remain in the same range, but the spatial distribution clearly changes. This indicates that both the electric and magnetic recordings reveal detailed information about the spatiotemporal distribution of neural activity in the slice.

### Effects of the width and thickness of the activated CA1 subarea on the peak magnetic fields

**Figure 4:**
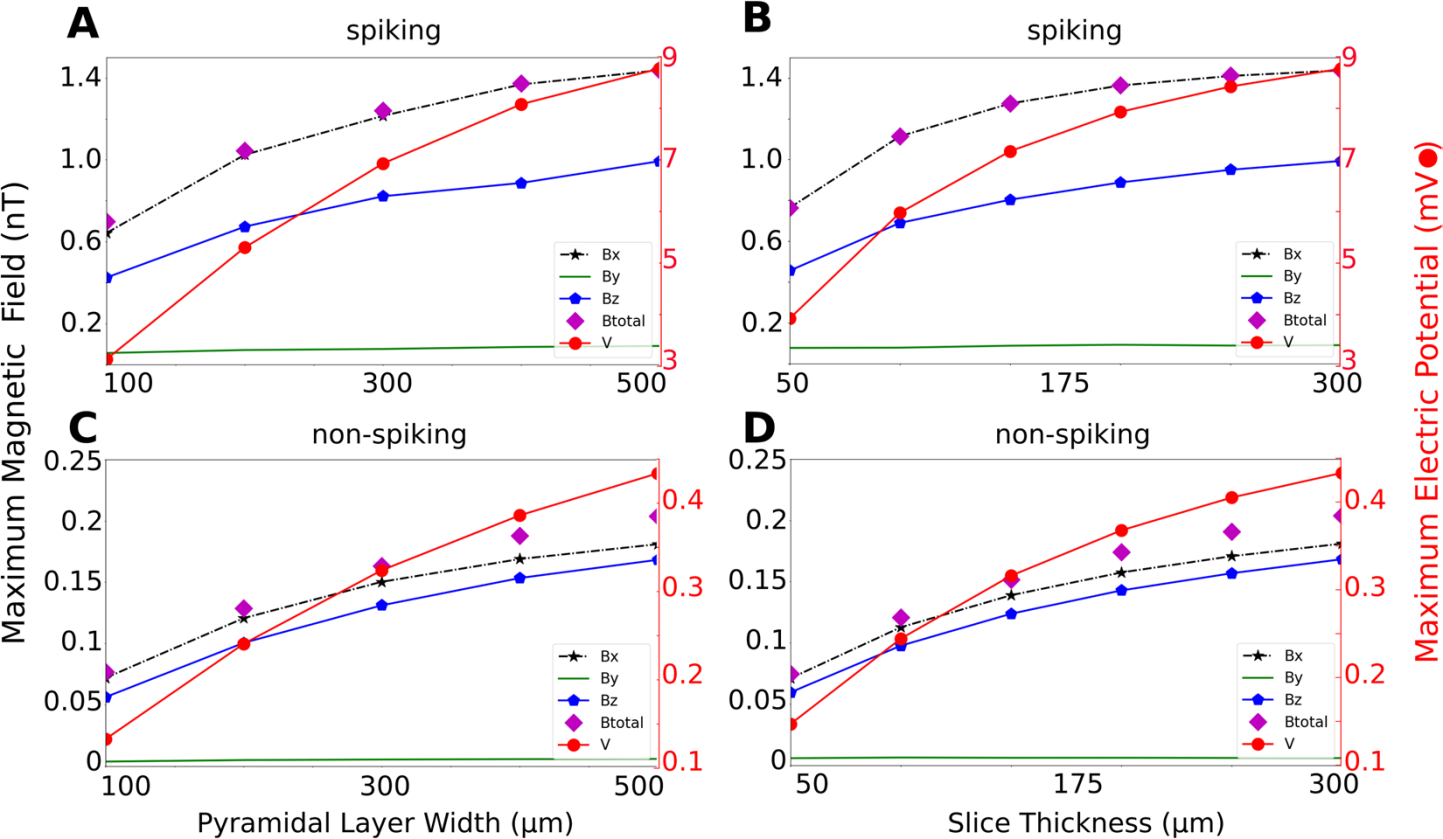
Dependence of the maximum field strength on the size of the active CA1 subarea. The distance of the active subarea to the sensor surface is 50 µm in all simulations. (**A)** Spiking case. The width of the subarea is varied from 100 µm to 500 µm, the thickness is 300 µm. (**B)** Spiking case. The thickness of the subarea is systematically varied from 50 µm to 300 µm, the width is 500 µm. (**C)** Non-spiking case. The width of the subarea (X direction in Fig. 1B) is varied from 100 µm to 500 µm, while the thickness is held constant at 300 µm. (**D)** Non-spiking case. The thickness of the subarea (Z direction in Fig. 1B) is systematically varied from 50 µm to 300 µm. The width is held constant at 500 µm.

Next, we characterized the dependence of the peak magnetic fields on the thickness of the hippocampus slice and the width of the activated subarea. When systematically increasing the width of the active region (X direction in Fig. 1B) while keeping a constant slice thickness of 300 µm, the peak magnetic field strength will start to saturate at a width of ~300 µm (Fig. 4A,C). Similarly, systematically increasing the slice thickness (Z direction in Fig. 1B) for a fixed width of 500 µm results in increasing peak magnetic fields that begin to saturate for a slice thickness of ~300 µm. For even thicker slices, the upper parts of the slice will not contribute markedly to the field measured by the sensor due to the steep decay of the magnetic field strength with distance to the neural sources.

The saturation is more clearly visible for the spiking vs. the non-spiking case. The reason is that the axially oriented current distributions that occur during the action potentials have the form of dipoles (Fig. 3B), causing a steeper decay in the field strength with increasing distance to the measurement site.

### Effect of temporal synchrony and synaptic strength on the magnetic signal strength

**Figure 5:**
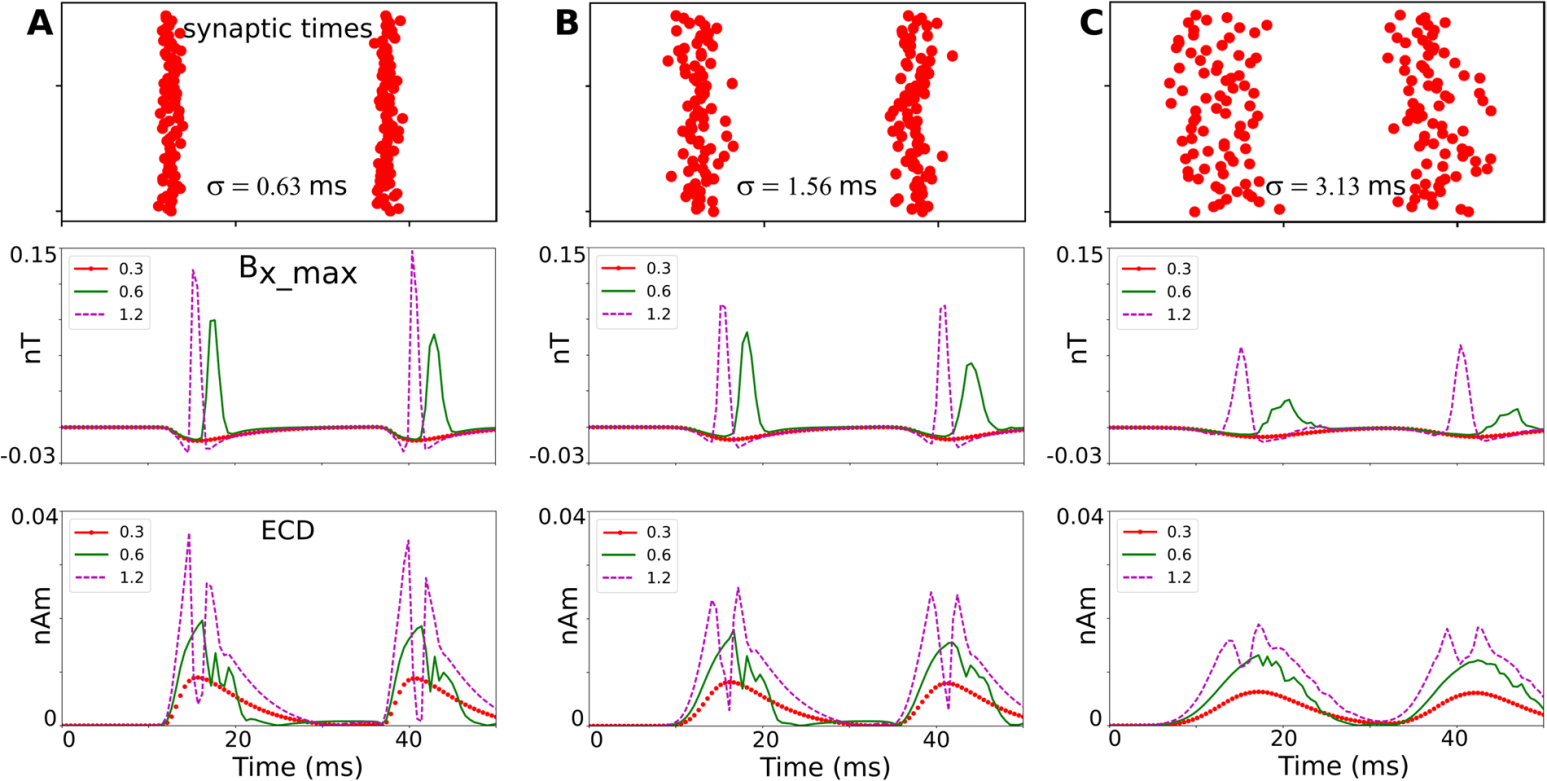
Dependence of the field strength on the temporal synchrony of the EPSPs and on the simulated strength of the glutamatergic synapses. A sublayer is simulated that contains 200 cells and has a width of 100 µm, a height of 50 µm, and a minimal distance of 150 µm to the diamond surface. The time points of the synaptic excitations are jittered in time using a Gaussian profile, determined by a standard deviation σ. The synaptic strengths were varied between 0.3 nS (resulting in non-spiking activity), 0.6 nS (resulting in just stable spiking activity) and 1.2 nS (resulting in more synchronous spiking). The top row depicts the temporal distribution of the synaptic excitations. The middle row shows the time course of the B_X_ component of the magnetic field, extracted at the spatial peak position. The bottom row shows the time course of the equivalent current dipole (ECD), again extracted at the spatial peak position. (**A)** Results for highly synchronous synaptic excitations (*σ* = 0.63 ms), (**B**) Synchronous synaptic excitations (*σ* = 1.56 ms) and, (**C)** Weakly synchronous excitations (*σ* = 3.13 ms).

In the above simulations of the spiking case, the synaptic strengths were chosen just high enough to activate all pyramidal cells in the subarea and the temporal jitter was chosen such that the duration of population spike in the LFPs was in the range of those seen in physiological slice recordings^30^. In the following, we aim to ensure the robustness of our results by testing how much the simulated peak magnetic fields depend on these choices. For completeness, we also report on the impact of the temporal jitter on the magnetic fields for the non-spiking case. In order to speed up the calculations and without loss of generality, these simulations were obtained for a small region containing 200 cells. While the peak field strength increases only moderately for highly synchronous input (second rows of Fig. 5A vs. B), it decreases around twofold when the input is less synchronous (second rows of Fig. 5C vs. B). Doubling the synaptic strength from 0.6 nS to 1.2 nS increases the peak magnetic fields for the spiking case in the range 33–50%, unless the synaptic events are highly asynchronous. Taken together, given the conservative estimates of the temporal synchrony and synaptic strength used for the spiking case in the main part of the results, in an experiment we might expect moderately higher peak magnetic field strengths than estimated here.

Since magnetic fields strongly depend on the neural axial current densities, we validated our calculations by determining an equivalent current dipole (ECD) by summing the resulting neural currents and comparing the simulated ECD strength with values reported for hippocampal pyramidal cells^31^. The ECD is given by $\overrightarrow{{{\rm{Q}}}_{{\rm{i}}}}=\sum _{{\rm{k}}}{{\rm{I}}}_{{\rm{i}}}^{{\rm{k}}}{\overrightarrow{{\rm{L}}}}^{{\rm{k}}},$ with L^k^ indicating the length of the k^th^ cylinder and ${{\rm{I}}}_{{\rm{i}}}^{{\rm{k}}}$ the axial current flow in the cylinder k at the time frame i^31^. Previously the ECD of a single cell is estimated as 0.2 pA-m when an AP occurs^8^. Accordingly, in our simulation of 200 highly synchronized neurons (Fig. 5A), in which the ECDs of the single neurons are simultaneous enough to add up linearly, the total ECD reaches up to 35 pA-m. The remaining small discrepancy may result from slightly different cell morphologies.

### Temporal bandwidth of the neural magnetic fields

**Figure 6:**
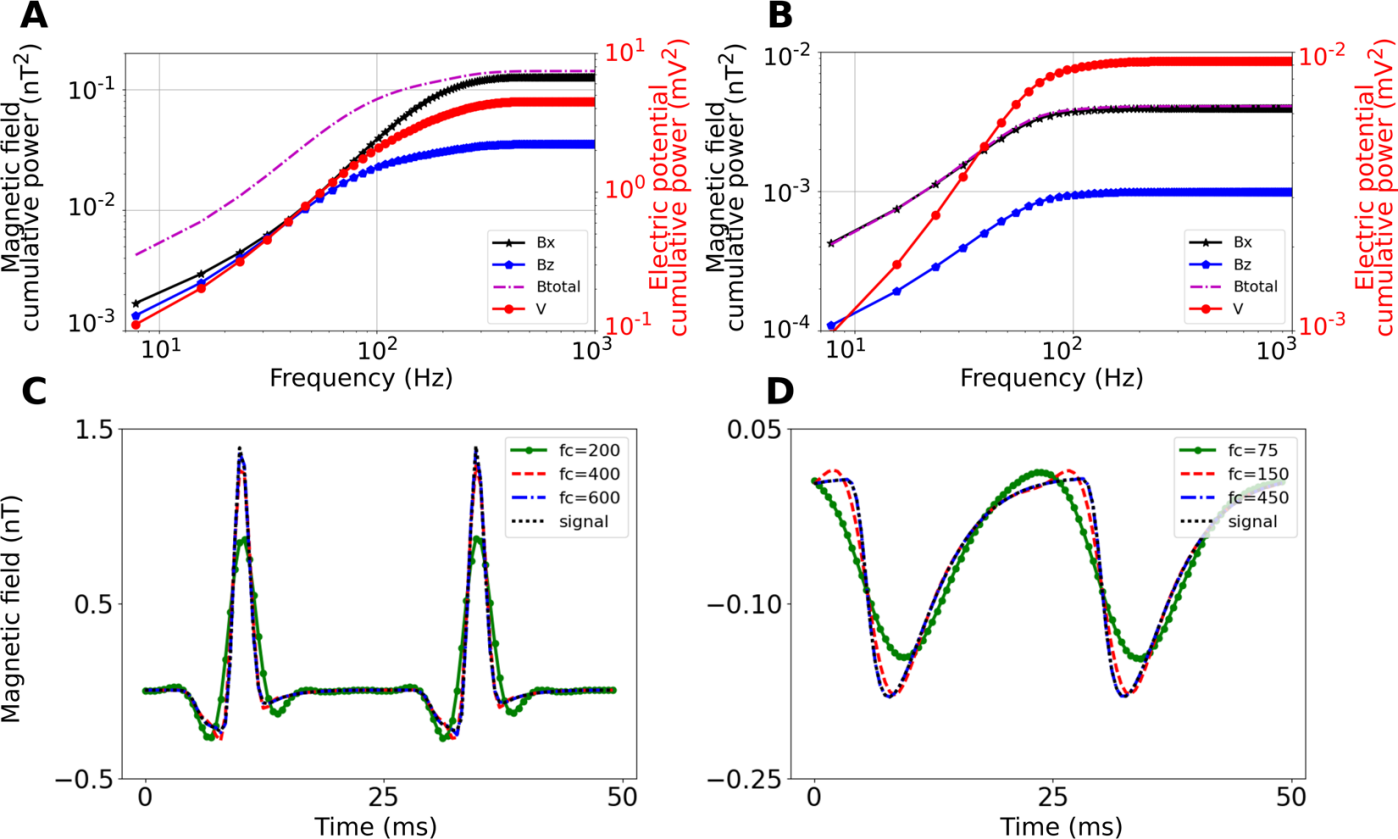
(**A)** Cumulative power of the magnetic field components, the total magnetic field and the electric potential for the spiking case of the 500 × 500 × 300 µm^3^ large CA1 subarea (50 µm distance to the sensor). (**B)** Cumulative power for the non-spiking case. Cumulative power of the LFP is shown for comparison. (**C**) Corresponding temporal waveforms for the spiking case, both non-filtered and low-pass filtered with a third-order Butterworth filter at different cut-off frequencies fc. A Butterworth filter was chosen due to its maximally flat magnitude response. The signal shape is well preserved for cut-off frequencies of 400 Hz and higher. (**D)** Temporal waveforms for the non-spiking case. The signal is well preserved for cut-off frequencies of 150 Hz and higher. The time distributions in (**C** and **D**) are extracted at the spatial point where the maximum field occurs.

For the spiking case, most of the signal power is still maintained when using a cut-off frequency of 400 Hz (Fig. 6A,C), suggesting a temporal sampling rate of 800 Hz according to the Nyquist criterion. For the non-spiking case, a cut-off frequency of 150 Hz still maintains most of the signal power (Fig. 6B,D), indicating that a sampling rate of 300 Hz is sufficient.

### Achievable 2D spatial resolution and required in-plane resolution and sensitivity of the sensor

**Figure 7:**
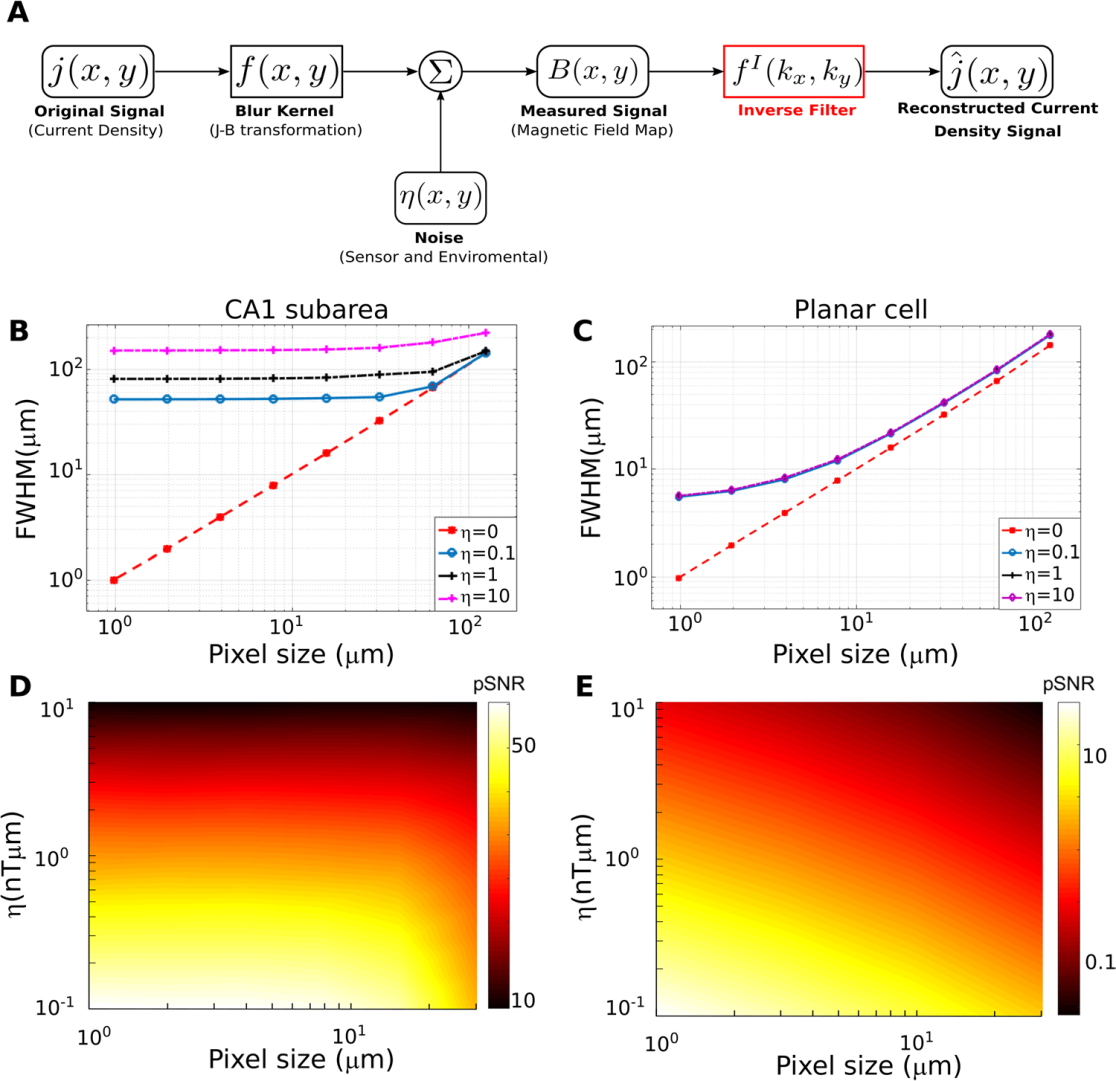
(**A)** Schematic outline of the reconstruction of neural axial current densities from the measured magnetic fields. The shown pipeline reconstructs the 2D projection of the current densities via an optimal Wiener deconvolution filter. (**B)** Recording from the CA1 subarea. Shown is the achievable 2D system resolution (characterized by the full width at half maximum – FWHM – of the reconstructed neural axial current density) in dependence on the in-plane resolution of the sensor. The neural source has a height of 300 µm in Z direction, starting at a distance of 50 µm from the diamond surface. It induces a constant current density J_Y_ in Y direction across its complete height to coarsely mimic the neural axial currents of pyramidal cells when the complete height of the slice is activated. The source strength was selected so that the magnetic peak field strength was 1.5 nT at the diamond surface, thereby matching the peak field strength observed in the simulations of the CA1 subarea. The achieved system resolution is almost constant for higher in-plane resolutions, but drops off quickly for too low in-plane resolutions. (**C**) Recording of a planar cell. Shown is the achievable system resolution in dependence on the in-plane resolution. The distance of the neural point source from the diamond surface is 1 µm and the length is 2 µm. The source strength was selected to yield a peak magnetic field of 2.5 nT at the diamond surface, which corresponds to the peak field around the soma (Fig. 2). Applying the results to others parts of the single planar pyramidal cell thus requires rescaling, as our neural simulations indicate peak magnetic fields of <1 nT for the latter. Except for very high in-plane resolutions of <4 µm, the system resolution depends linearly on the in-plane resolution. (**D)** Peak signal-to-noise ratio (pSNR) for recordings from the CA1 subarea as a function of the in-plane resolution of the sensor (pixel size) and the signal-to-noise power of the source, η. As long as the in-plane resolution is chosen high enough to maintain the best possible system resolution, also the pSNR of the reconstructed axial current densities remains constant. (**E)** Peak-signal-to-noise ratio (pSNR) for recordings from a planar cell.

In order to characterize the achievable spatial resolution and its dependence on the system parameters, we simulated the magnetic field of a point-like axial current density and reconstructed its 2D projection via an optimal Wiener deconvolution filter (see Methods). A schematic of the signal analysis pathway is presented in Fig. 7A. We report the point spread function of the reconstructed signal to characterize the achievable 2D spatial resolution and the peak signal-to-noise ratio (pSNR)^32^ of the reconstructed signal, which is defined as ratio of the peak signal to the mean square error of the noise (i.e. the noise standard deviation). We systematically tested the dependence of these metrics on the in-plane resolution and the area-normalized noise level η of the system (i.e. the noise level per unit area for the chosen sampling frequency).

**Figure 8:**
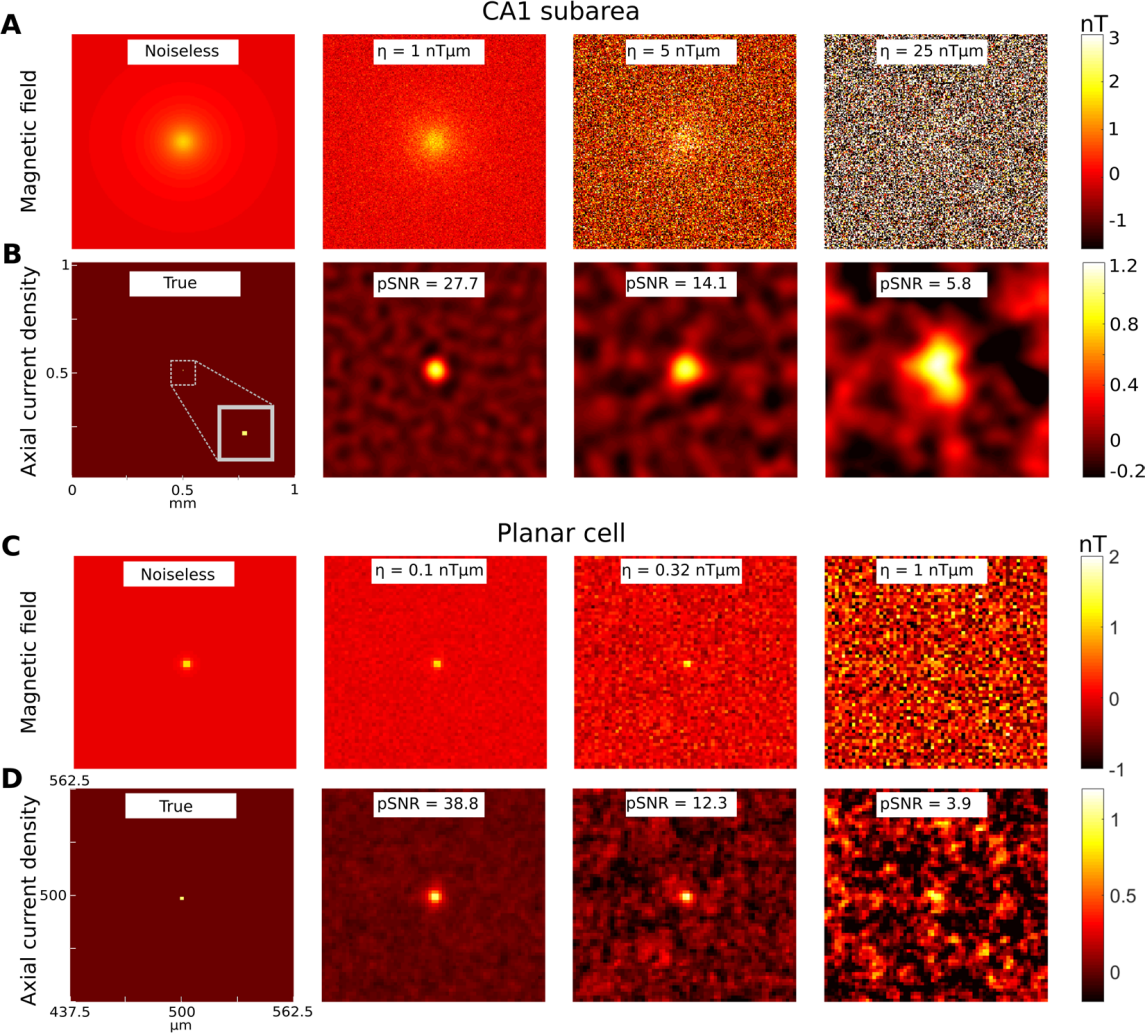
Examples of reconstructed neural axial current densities at varying pSNR levels. (**A)** Recording from the CA1 subarea. A neural point source with 50 µm distance to the diamond plane and a length of 300 µm oriented in Z direction is simulated. It injects a constant current density J_Y_ in Y direction across its complete height. The strength of the source is chosen so that a peak magnetic field strength of 1.5 nT is reached in the sensor plane. The in-plane resolution of the sensor is set to 7.8 µm (128 × 128 pixels in 1 mm^2^). The B_X_ component of the measured magnetic field is shown for the noiseless case and for three different levels of the signal-to-noise power of the recorded magnetic field. (**B)** The normalized axial current densities reconstructed from the corresponding magnetic fields shown in (**A**). Each plot is normalized to the maximum of its noiseless reconstruction with the same spatial filter settings. (**C**) Recording of a planar cell. A neural point source with 1 µm distance to the diamond and a length of 2 µm is simulated. Its strength is chosen to give a peak magnetic field strength of 2.5 nT in the sensor plane. The B_X_ component of the measured magnetic field is shown for the noiseless case and for three different levels of the signal-to-noise power. The in-plane resolution of the sensor is set to 2 µm (512 × 512 pixels in 1 mm^2^) and a field of view of 125 × 125 µm^2^ is selected for better visualization. Since the magnetic field is very localized, the “recorded” peak magnetic field strength is slightly lower than 2.5 nT due to spatial averaging in the 2 × 2 µm areas of the sensor pixels. (**D**) The normalized axial current densities reconstructed from the corresponding magnetic fields in (**C**). Each plot is normalized to the maximum of its noiseless reconstruction with the same spatial filter settings.

A one-dimensional source injecting currents in Y direction, and with a length of 300 µm in Z direction and at a distance of 50 µm perpendicular to the sensor surface was used to characterize the application scenario of recording from a CA1 subarea. In that case, the pixel size should be not much larger than 10 µm in order to maintain the best possible resolution of the reconstructed signal (Fig. 7B) for the range of tested noise levels η. Increasing the pixel size clearly beyond 10 µm will also tend to decrease the pSNR of the reconstructed axial current density (Fig. 7D). Figure 8A,B show examples of the magnetic field and reconstructed axial current density at different noise levels. A reasonable reconstruction requires a pSNR >10, which is exceeded at system noise levels lower than η = 10 nTµm. For control, the analyses was repeated for a source with 50 µm length in Z direction (i.e., from z = 50 µm to 100 µm; Fig. S14). As expected, the achievable 2D resolution is slightly lower for the same noise level, as the total source strength is reduced to 1/6.

The analysis was also performed for a source with 1 µm distance to the diamond and a length of 2 µm to characterize the required system parameters when recording from a planar pyramidal cell (Figs 7C,E and 8C,D). Here, the pixel size should not be larger than 2 µm in order to maintain the best possible resolution of the reconstructed signal (Fig. 7C). Increasing the pixel size beyond 2 µm will also decrease the pSNR of the reconstructed axial current density (Fig. 7E) and in general, system noise levels < ~0.4 nTµm are required to achieve a pSNR > 10.

For further demonstration of the performance of the reconstruction pipeline, we compared the reconstructed current densities at different SNR levels with the underlying true axial current distributions for the cases of the CA1 subarea and planar pyramidal cell (Fig. S15; see chapter S7 of the Supplementary Material for details). As expected, the results indicate that a good reconstruction of the true current density requires similar noise levels as determined from the corresponding point spread functions.

## Discussion

We have characterized the spatiotemporal characteristics of neural magnetic fields of brain slices through comprehensive computational simulations and theoretical analyses. Our approach encompassed the use of a pyramidal cell model with a realistic morphology and dynamics, FEM simulations to test for the impact of return currents, tissue anisotropy and boundary conditions, tests for the impact of changes in synaptic strength and synchrony of the input, and systematic analyses of the required sensitivity levels and expected spatial resolution. Our results suggest that peak magnetic field strengths of ~1.5 nT can be expected in an experiment, and that the temporal shape is preserved when sampling at a frequency of 800 Hz or higher. The achievable spatial resolution is ~100 µm for η = 10 nTµm, which is the highest noise level of the imaging system that still allows for acceptable axial current density reconstructions, and it improves with decreasing system noise levels. An in-plane resolution of the system of around 10–20 µm should be chosen to achieve the best resolution and SNR of the reconstructed axial current density distributions. This corresponds to an array size of around 100 × 100 pixels for a 1 × 1 mm² imaging area, which is easily feasible using suited cameras^33^.

The suggested optomagnetic approach is an interesting alternative to MEA and VSDI methods. While wide-field VSDI can display propagating network activity across the sensor in anatomically larger network, this is at the cost of resolution compared to the recording of current sources and sinks, as only voltage changes are registered. The temporal resolution of VSDI depends on choice of the dye, of which some exhibit weak toxicity, thus also potentially limiting life-time of the slice^34^. Compared to MEAs, optomagnetic recordings do not suffer from 1/f noise at the electrode-electrolyte interface and should be well suited for low LFP frequencies (likely requiring some shielding from ambient fields). They provide complementary information, as the magnetic field represents mainly the axial neural currents, while the extracellular potential recorded by MEAs is sensitive to the membrane currents. Our FEM simulations (Figs S4 and S9) indicate that the magnetic field depends less on the properties of the extracellular volume conductor than the electric potential. For B_X_, a rather simple model of an infinite half-space seems sufficient for accurate forward simulations, while no model is needed for B_Z_. This suggests that optomagnetic recordings should be well suited for accurate source reconstructions when a sufficient SNR level has been established. The approach also offers the possibility to measure both the B_X_ and B_Z_ component in quick succession by switching the background gradient field direction, further supporting accurate localization. Finally, the measured “pixel size” is determined by the optics between the diamond and the camera, so that future setups could also flexibly change their field-of-view by adapting the optics, similar to microscopes.

Our simulations rely on some uncertain parameters and simplifying assumptions that might affect the estimated peak magnetic fields and the achievable 2D resolution. However, we are confident that their accuracy is good enough in order not to hamper their value to guide further methods development. For example, our simulations assume that all pyramidal cells in the CA1 subarea are recruited by the stimulus, which might suggest higher peak field strengths than occurring in reality. On the other hand, we assume a cell density of 100 cells in a 50 × 50 × 50 µm^3^ volume, which is a conservatively chosen lower limit and is based on the sparse data that is available for the cell density^35,36^. While this indicates that our results might underestimate the peak field strengths, it should be noted that they can be simply linearly rescaled if required. We have also shown that similar estimates for the achievable 2D system resolution are obtained when not the complete height of the slice is activated (Figs 7 and S14). The synchrony of the synaptic events caused by the electrical stimulation was conservatively estimated, again tending to decrease the peak magnetic fields. A higher synchrony will increase the peak fields, but will also require to sample at higher frequencies, suggesting that both effects will approximately “cancel out”. Our assumption of a 50 µm thick layer of fully dysfunctional cells closest to the diamond sensor is a conservative choice that results in an underestimation of the peak field strengths and the achievable resolution. Specifically, electrophysiological recordings with single electrodes show that intact pyramidal cell activity can be found already within this layer. In a related manner, it is worth noting that the incorporated scale factor for B_X_ to account for the impact of the extracellular current flow neglects that also the distribution of B_X_ changes slightly (chapter S3 of the Supplementary Material). Specifically, it gets “narrower” in X direction, suggesting that the achievable resolution is higher than given by our estimates. As a guidance for further development of the measurement approach, our analyses focused on the achievable 2D resolution in dependence on the system “pixel size” and noise levels. Extending the results presented here to explore the feasibility of full 3D reconstructions of the axial current density would be an interesting topic for future studies^37^.

In contrast to imaging the magnetic fields of a brain slice, our results show that imaging of a planar neuron placed on the sensor surface poses more demanding requirements on the system (Fig. 7C and E). This is despite higher predicted peak fields occurring close to the soma, and is a result of the spatially more confined magnetic fields of the single neuron (please note that we ignored the effects of the extracellular volume conductor for the planar neuron case, which might potentially decrease the peak field). The latter property allows for imaging at very high spatial resolutions below 10 µm, but only when very low system noise levels (η < 0.4 nTµm) can be achieved. In contrast to the reconstruction from the spatially smoother neural magnetic fields expected for a brain slice, noise suppression by spatial integration of information as effectively occurring during Wiener deconvolution, is far less feasible in case of a single planar neural cell.

When diamond sensors are used for sensing low-frequency (DC to kHz) magnetic fields, their sensitivity is fundamentally limited by the NV ensemble density, *n*_*NV*_, the dephasing time, ${T}_{2}^{\ast }$, and the on-resonance fluorescence contrast, *C*^1,3^. The volume and bandwidth normalized sensitivity limit can be expressed as

1 $${\eta }_{V}\cong \frac{h}{g{\mu }_{B}}\frac{1}{C\sqrt{\epsilon \,{n}_{NV}{T}_{2}^{\ast }\,}},$$

where $\frac{g{\mu }_{B}}{h}\cong 28$ Hz/nT is the NV gyromagnetic ratio, and ${\epsilon } \sim {10}^{-2}$ is the photon collection efficiency. While this expression is valid for pulsed measurements, continuous wave (CW) techniques typically show a reduced sensitivity due to power broadening. CW techniques, however, are easier to implement in a wide-field imaging setup due to reduced technical requirements. Recently, a volume normalized sensitivity of ${\eta }_{V}=34\,{\rm{nT}}\mu {{\rm{m}}}^{\frac{3}{2}}{{\rm{Hz}}}^{-\frac{1}{2}}$ has been demonstrated using a CW protocol and isotopically engineered diamond with *n*_*NV*_ ~ 1 ppm and ${T}_{2}^{\ast } \sim 0.5$ µs^10^. At this level of sensitivity, imaging of the hippocampus tissue with a 5 µm-thick sensing layer and a sampling rate of 1 kHz corresponds to an area-normalized sensitivity of around 480 nTµm (see Methods eq. (9)). Imaging thus requires averaging of around 2400 trials in order to achieve an area-normalized sensitivity of 10 nTµm of the averaged signal. Further improvement is anticipated from advances in diamond preparation techniques that would lead to a much longer dephasing time, ${T}_{2}^{\ast } \sim 20$ µs at a similar NV concentration^10^. In order to benefit from such slow-dephasing samples, power broadening has to be avoided and CW protocols can no longer be used. An overall sensitivity improvement of more than two orders of magnitude is expected from combining improved diamond samples with a Ramsey-type measurement scheme^10^. This would allow for planar cell imaging with averages of around 10^2^–10^3^ trials, while neural activity in brain slices would already be detectable in a single shot. While the integration of the NV diamond sensor into a functional setup poses additional challenges, our simulation results indicate that the approach suggested here has the potential to reveal important insights into the neural network dynamics of brain slices.

## Methods

### Forward Modeling Scheme for Calculation of the Neural Magnetic Field and Electric Potential

The simulations of the extracellular neural magnetic fields and electric potential proceeds in two steps. First, the membrane potentials ${{\rm{V}}}_{{\rm{m}}}^{{\rm{n}}}\,\,$and transmembrane currents ${{\rm{I}}}_{{\rm{m}}}^{{\rm{n}}}\,\,$of the simulated neurons are calculated using the NEURON software package (v7.4)^20^. For that, NEURON solves the cable equation for complex neural structures, which are discretized into multiple compartments with nonlinear ion channels, and the discretized intracellular and extracellular regions are represented by axial resistances connected by membrane networks^38^. This is schematically depicted in the core-conductor model in Fig. 1C. Then, the extracellular magnetic fields, **B**, and the electric potential, Φ_e_, are determined from the membrane potentials, ${{\rm{V}}}_{{\rm{m}}}^{{\rm{n}}}$, and transmembrane currents, ${{\rm{I}}}_{{\rm{m}}}^{{\rm{n}}}$, using the forward modelling scheme:

2 $${\bf{B}}({\bf{r}},{\rm{t}})=\frac{{{\rm{\mu }}}_{0}}{4{\rm{\pi }}}\sum _{{\rm{n}}=1}^{{\rm{N}}}\frac{{{\bf{I}}}_{{\rm{axial}}}^{{\rm{n}}}({\rm{t}})\times {\hat{{\rm{\rho }}}}_{{\rm{n}}}}{{{\rm{\rho }}}_{{\rm{n}}}}[\frac{{{\rm{h}}}_{{\rm{n}}}}{\sqrt{{{\rm{h}}}_{{\rm{n}}}^{2}\,+{{\rm{\rho }}}_{{\rm{n}}}^{2}}}-\frac{{{\rm{l}}}_{{\rm{n}}}}{\sqrt{{{\rm{l}}}_{{\rm{n}}}\,+{{\rm{\rho }}}_{{\rm{n}}}^{2}}}]$$

3 $${\varphi }_{{\rm{e}}}({\bf{r}},{\rm{t}})=\frac{1}{4\pi \sigma }\sum _{{\rm{n}}=1}^{{\rm{N}}}\frac{{{\rm{I}}}_{{\rm{m}}}^{{\rm{n}}}({\rm{t}})}{{{\rm{\Delta }}s}_{{\rm{n}}}}\,{\rm{l}}{\rm{o}}{\rm{g}}|\frac{\sqrt{{{\rm{h}}}_{{\rm{n}}}^{2}+{\rho }_{{\rm{n}}}^{2}}-{{\rm{h}}}_{{\rm{n}}}}{\sqrt{{{\rm{l}}}_{{\rm{n}}}^{2}+{\rho }_{{\rm{n}}}^{2}}-{{\rm{l}}}_{{\rm{n}}}}|$$

with ${{\rm{I}}}_{{\rm{axial}}}^{{\rm{n}}}=\frac{{{\rm{V}}}_{{\rm{m}}}^{{\rm{n}}}-{{\rm{V}}}_{{\rm{m}}}^{{\rm{n}}-1}}{{{\rm{\Delta }}{\rm{s}}}_{{\rm{n}}}{{\rm{r}}}_{{\rm{i}}}^{{\rm{n}}}}$. It is assumed that the neuron is divided into N compartments and Δs_n_ denotes the length of each compartment. In addition, ρ_n_ represents the distance perpendicular to the orientation of the main axis of the compartment, h_n_ the longitudinal distance from the end of the compartment, and l_n_ = h_n_ + Δs_n_ the longitudinal distance from the start of the compartment^39^. The extracellular potentials are determined using the LFPy toolbox^40^ and the magnetic field is calculated by adapting LFPy to evaluate Eq. (2). For the simulations of the brain slice, the B_X_ component determined via Eq. (2) is additionally corrected by a scaling factor to account for the impact of the extracellular current flow (Suppl. Material chapter S3). This factor was empirically determined using FEM (Finite-Element Method) simulations of the magnetic field of a simple neural structure (a straight axon) in dependence of its distance to the diamond surface. While the high number and histological complexity of the pyramidal neurons renders a direct assessment of the extracellular current flow via FEM infeasible for the main scenario, this strategy approximately accounts for its impact on the results. The B_Z_ component was found to be far less affected in the FEM simulations, and is thus not corrected. The FEM results revealed an expected strong impact of the extracellular volume conductor on the electric potential at the diamond surface. However, when allowing for a thin “leakage” layer filled with saline between the hippocampus slice and the surface, the strength of the electric potential was very similar to the strength directly estimated via Eq. (3). Therefore, also the electric potential is not corrected. Further details on the forward modeling scheme are summarized in the Supplementary Material, which also covers the validation of our simulation approach by comparing it with previously presented *in vitro* results^10,41^. The employed forward scheme was additionally validated by comparison with the numerical solution obtained via FEM for the case of a long straight axon, which is also summarized in the Supplementary Material.

The total magnetic field and electric potential created by activation of the CA1 subarea are determined by calculating the field for each contained pyramidal cell, and the final result is obtained by a linear superposition of the results of the single cells. For that, the 500 µm × 500 µm × 300 µm large CA1 subarea is divided into several layers with 50 µm thickness along the Z direction, and 1000 pyramidal cell models are placed in each layer^35,36^, with the main dendrites pointing in the Y direction. The soma locations are randomly jittered within the layer, with the jitter being restricted to a 50 µm wide band in Y direction. As the same cell model was used for all locations, the cells are rotated randomly around the orientation of their main dendrite in order to prevent the formation of artefacts created by the field superposition of the single cells. In the literature, the neural density of pyramidal cells is given either as total number (4 × 10^5^ cells in CA1, corresponding to 2.25 × 10^6 ^cells/mm^3^)^35^ or as density in the pyramidal cell layer (1.74×10^6^ cells/mm^3^)^36^. These numbers suggest 281 cells^36^ and 217 cells^35^ in a 50 × 50 × 50 µm volume, based on the assumption that the soma of the pyramidal cells are contained in a 50 µm wide band in Y direction, denoted as the “pyramidal layer”. The experimental studies did not distinguish different neural cell types in the pyramidal layer when determining the cell counts, and while pyramidal cells are the most frequent cell type in that layer, using the above results without correction would overestimate the number of pyramidal cells. We therefore base our simulations on 100 cells in a 50 × 50 × 50 µm volume, which is a conservative lower limit. However, the neuronal density varies for different slices and species and, if desired, our results can be easily linearly rescaled to match those for a different neuron density. We consider only pyramidal cells for our simulations, as their regular and longitudinal cell morphologies result in well detectable external fields which sum up across neighboring cells. The external magnetic fields and electric potential of layered cortical structures are thus dominated by the responses of this neural cell type^29^.

### Achievable spatial resolution and required in-plane resolution and sensitivity of the sensor

The reconstruction of the neural axial current density (representing the current flow within the neurons) in the brain slice from the measured 2D magnetic field distributions allows us to determine the spatial position and extent of the activated hippocampal subarea. In the following, we focus on the reconstruction of the 2D projection of the field on the diamond plane rather than a full 3D reconstruction. This helps to limit the complexity of the analysis, while still giving useful information about the system parameters. The current density reconstruction then has the form of a 2D deconvolution problem, for which Wiener deconvolution is the optimal method in case of additive noise^32^. It applies an inverse Wiener filter in the spatial frequency domain to “undo” the effects of the magnetic field that acts as a spatial low-pass filter of the neural axial current density (Fig. 7A). We are interested to determine the maximally achievable spatial resolution and the signal-to-noise ratio of the reconstructed current density. In particular, in order to guide the development of the measurement setup, we aim to characterize their dependence on the in-plane resolution of the system and on its signal-to-noise ratio.

In the following, we consider the magnetic field of a 2D point source and determine the full width at half maximum (FWHM) and the peak-signal-to-noise ratio (pSNR) of the current density reconstructed by inverse Wiener filtering. Given the orientation of the pyramidal cells along the Y direction in the modelled CA1 subarea (Fig. 1B), the J_X_ and J_Z_ components of the neural axial current densities are small and their contribution to the magnetic field can be neglected. The neural source currents J_Y_ result in a magnetic field distribution at the diamond surface that has only B_X_ and B_Z_ components. We focus on the B_X_ component of the field as it has higher peak strengths (Fig. 4). In addition, we assume a homogenous excitation of the pyramidal cells along the full height of the CA1 subarea (except for the layer of dead cells), so that the strength of the neural axial current density J_Y_ is constant across the depth of the tissue. Denoting the thickness of the layer of dead cells by z_0_ and the thickness of the layer of active cells as d, the B_X_ component of the magnetic field at the diamond surfaces is:

4 $${{\rm{B}}}_{{\rm{X}}}({\rm{x}},{\rm{y}})=\frac{{\mu }_{0}}{4\pi }{\int }_{0\,}^{{\rm{d}}\,}{\int }_{-{\rm{\infty }}}^{{\rm{\infty }}}{\int }_{-{\rm{\infty }}}^{{\rm{\infty }}}\frac{s({{\rm{z}}}_{0}+{{\rm{z}}}^{{\rm{^{\prime} }}}){{\rm{J}}}_{{\rm{y}}}({{\rm{x}}}^{{\rm{^{\prime} }}},{{\rm{y}}}^{{\rm{^{\prime} }}})({{\rm{z}}}_{0}+{{\rm{z}}}^{{\rm{^{\prime} }}})}{{[{({\rm{x}}-{{\rm{x}}}^{{\rm{^{\prime} }}})}^{2}+{({\rm{y}}-{{\rm{y}}}^{{\rm{^{\prime} }}})}^{2}+{({{\rm{z}}}_{0}+{{\rm{z}}}^{{\rm{^{\prime} }}})}^{2}]}^{\frac{3}{2}}\,}\mathrm{dx}{\rm{^{\prime} }}\mathrm{dy}{\rm{^{\prime} }}\mathrm{dz}{\rm{^{\prime} }}.$$

Function *s* denotes a rational polynomial $s(d)=({{\rm{a}}}_{1}+\frac{{{\rm{a}}}_{2}}{d+c})$, which is included to correct for the effects of the finite extracellular volume conductor on B_X_ (see Fig. S8A and the corresponding chapter 3 of the Supplementary Material). Transformation to the spatial frequency domain and integration along z yields the following simplified relation^42^:

5 $$\begin{array}{rcl}{{\rm{b}}}_{{\rm{x}}}({{\rm{k}}}_{{\rm{x}}},{{\rm{k}}}_{{\rm{y}}}) & = & [{{\rm{a}}}_{1}{{\rm{\mu }}}_{0}\,\exp (-({{\rm{z}}}_{0}+\frac{{\rm{d}}}{2})\sqrt{({{\rm{k}}}_{{\rm{x}}}^{2}+{{\rm{k}}}_{{\rm{y}}}^{2})})\frac{\sinh (\frac{{\rm{d}}}{2}\sqrt{({{\rm{k}}}_{{\rm{x}}}^{2}+{{\rm{k}}}_{{\rm{y}}}^{2})})}{\sqrt{({{\rm{k}}}_{{\rm{x}}}^{2}+{{\rm{k}}}_{{\rm{y}}}^{2})}}\\  &  & +\,\frac{{{\rm{a}}}_{2}{{\rm{\mu }}}_{0}}{2}{{\rm{E}}}_{1}(({{\rm{z}}}_{0}+{\rm{c}})\sqrt{({{\rm{k}}}_{{\rm{x}}}^{2}+{{\rm{k}}}_{{\rm{y}}}^{2})})\\  &  & -\,\frac{{{\rm{a}}}_{2}{{\rm{\mu }}}_{0}}{2}{{\rm{E}}}_{1}(({{\rm{z}}}_{0}+{\rm{c}}+{\rm{d}})\sqrt{({{\rm{k}}}_{{\rm{x}}}^{2}+{{\rm{k}}}_{{\rm{y}}}^{2})})]\,{{\rm{j}}}_{{\rm{y}}}({{\rm{k}}}_{{\rm{x}}},{{\rm{k}}}_{{\rm{y}}})\\  & = & {\rm{f}}({{\rm{k}}}_{{\rm{x}}},{{\rm{k}}}_{{\rm{y}}}){{\rm{j}}}_{{\rm{y}}}({{\rm{k}}}_{{\rm{x}}},{{\rm{k}}}_{{\rm{y}}}),\end{array}$$

where k_x_ and k_y_ are spatial frequencies and E_1_ is the exponential integral. Equation (5) shows that the magnetic field acts as a spatial low-pass filter of the axial current density. Its cut-off frequency decreases with increasing distance to the sensor and increasing slice thickness. Considering that the magnetic field stated in eq. (5) is the convolution function of the system (or source-to-measurement transformation filter), the Wiener deconvolution filter is given by^32^

6 $${{\rm{f}}}^{{\rm{I}}}({{\rm{k}}}_{{\rm{x}}},{{\rm{k}}}_{{\rm{y}}})=\frac{\bar{{\rm{f}}}({{\rm{k}}}_{{\rm{x}}},{{\rm{k}}}_{{\rm{y}}})}{{|{\rm{f}}({{\rm{k}}}_{{\rm{x}}},{{\rm{k}}}_{{\rm{y}}})|}^{2}+\frac{{{\rm{s}}}_{{\rm{\eta }}}({{\rm{k}}}_{{\rm{x}}},{{\rm{k}}}_{{\rm{y}}})}{{{\rm{s}}}_{{\rm{j}}}({{\rm{k}}}_{{\rm{x}}},{{\rm{k}}}_{{\rm{y}}})}},$$

with the overbar indicating the complex conjugate, s_j_ denoting the power spectral density (PSD) of the axial current density and s_η_ the PSD of the noise. The ratio between s_η_ and s_j_ controls the amount of regularization that is applied to the deconvolution.

As a next step, we assume a point source with known signal power, and relate the ratio between s_η_ and s_j_ to this signal power and to known system parameters. Assuming spatially uncorrelated white Gaussian noise, s_η_ is constant across spatial frequencies. The noise power of the image is then given as

7 $${{\rm{\sigma }}}_{{\rm{n}}}^{2}=\frac{1}{{(2{\rm{\pi }})}^{2}}{\int }_{-{{\rm{k}}}_{{\rm{sy}}}}^{{{\rm{k}}}_{{\rm{sy}}}}{\int }_{-{{\rm{k}}}_{{\rm{sx}}}}^{{{\rm{k}}}_{{\rm{sx}}}}{{\rm{s}}}_{{\rm{\eta }}}{{\rm{dk}}}_{{\rm{x}}}{{\rm{dk}}}_{{\rm{y}}}=\frac{{{\rm{s}}}_{{\rm{\eta }}}{{\rm{k}}}_{{\rm{sx}}}{{\rm{k}}}_{{\rm{sy}}}}{{{\rm{\pi }}}^{2}},$$

where k_sx_, k_yx_ are the maximum spatial frequencies in dependence of the in-plane resolution of the image (k_sx_ = π/Δ_x_; k_sy_ = π/Δ_y_; Δ_x_ = Δ_y_ = Δ).

The noise power of the image can also be written in terms of the pixel-normalized noise level η_pixel_, the number of pixels MxM (for a 2D grid with M pixels in each direction) and the pixel area Δ^2^ as

8 $${{\rm{\sigma }}}_{{\rm{n}}}^{2}=\sum _{{\rm{j}}=1}^{{\rm{M}}}\sum _{{\rm{i}}=1}^{{\rm{M}}}{{\rm{\eta }}}_{{\rm{pixel}}}^{2}{{\rm{\Delta }}}^{2}={{\rm{\eta }}}_{{\rm{pixel}}}^{2}{{\rm{\Delta }}}^{2}{{\rm{M}}}^{2},$$

with

9 $${{\rm{\eta }}}_{{\rm{pixel}}}=\frac{{{\rm{\eta }}}_{{\rm{V}}}}{\sqrt{{{\rm{V}}}_{{\rm{pixel}}}}}\sqrt{{{\rm{f}}}_{{\rm{s}}}}=\frac{{{\rm{\eta }}}_{{\rm{V}}}}{{\rm{\Delta }}\sqrt{{\rm{h}}}}\sqrt{{{\rm{f}}}_{{\rm{s}}}}=\frac{{\rm{\eta }}}{{\rm{\Delta }}},$$

where f_s_ is the sampling frequency, and V_pixel_ the volume and h the height of the NV layer, respectively. Equation (9) relates the pixel-normalized noise level η_pixel_ to the volume-normalized sensitivity η_V_, which is usually reported to characterize the sensitivity of the system (see e.g.^10^). It further relates it to the area-normalized noise level η (i.e. noise level per unit area for the chosen sampling frequency), which we define to account for the fact that the height of the NV layer and the sampling frequency were already selected and are kept constant. Both η_V_ and η are independent of the chosen in-plane resolution, and they can be determined via measurements. Equations (7–9) can then be used to determine s_η_ from the area-normalized noise level η of the system:

10 $${{\rm{s}}}_{{\rm{\eta }}}={{\rm{\eta }}}^{2}{{\rm{M}}}^{2}{{\rm{\Delta }}}^{2}={{\rm{\eta }}}^{2}{{\rm{A}}}_{{\rm{FoV}}}.$$

If j_Y_(x, y) is assumed to be a single dipole source having a dipole moment σ_j_, its source power spectral density will be:

11 $${{\rm{s}}}_{{\rm{j}}}({{\rm{k}}}_{{\rm{x}}},{{\rm{k}}}_{{\rm{y}}})={|{\rm{FT}}\{{{\rm{j}}}_{{\rm{y}}}({\rm{x}},{\rm{y}})\}|}^{2}={{\rm{\sigma }}}_{{\rm{j}}}^{2}.$$

Incorporating these results into the Wiener deconvolution filter (eq. (6)) yields:

12 $${{\rm{f}}}^{{\rm{I}}}({{\rm{k}}}_{{\rm{x}}},{{\rm{k}}}_{{\rm{y}}})=\frac{\bar{{\rm{f}}}({{\rm{k}}}_{{\rm{x}}},{{\rm{k}}}_{{\rm{y}}})}{{|{\rm{f}}({{\rm{k}}}_{{\rm{x}}},{{\rm{k}}}_{{\rm{y}}})|}^{2}+\frac{{{\rm{\eta }}}^{2}{{\rm{A}}}_{{\rm{FoV}}}}{{{\rm{\sigma }}}_{{\rm{j}}}^{2}}},$$

which indicates that the filter expression is independent of the chosen pixel size. Applying the Wiener filter to reconstruct the current density of the point source for a given pixel size Δ finally yields:

13 $$\begin{array}{rcl}\hat{{\rm{j}}}({\rm{x}},{\rm{y}}) & = & \frac{1}{{(2{\rm{\pi }})}^{2}}{\int }_{-{{\rm{k}}}_{{\rm{sy}}}}^{{{\rm{k}}}_{{\rm{sy}}}}{\int }_{-{{\rm{k}}}_{{\rm{sx}}}}^{{{\rm{k}}}_{{\rm{sx}}}}{{\rm{\sigma }}}_{{\rm{j}}}{{\rm{f}}}^{{\rm{I}}}({{\rm{k}}}_{{\rm{x}}},{{\rm{k}}}_{{\rm{y}}}){\rm{f}}({{\rm{k}}}_{{\rm{x}}},{{\rm{k}}}_{{\rm{y}}})\,{{\rm{e}}}^{{\rm{i}}({{\rm{k}}}_{{\rm{x}}}{\rm{x}}+{{\rm{k}}}_{{\rm{y}}}{\rm{y}})}{{\rm{dk}}}_{{\rm{x}}}{{\rm{dk}}}_{{\rm{y}}}\\  & = & \frac{1}{{(2{\rm{\pi }})}^{2}}{\int }_{-\infty }^{\infty }{\int }_{-\infty }^{\infty }({{\rm{\sigma }}}_{{\rm{j}}}{{\rm{f}}}^{{\rm{I}}}({{\rm{k}}}_{{\rm{x}}},{{\rm{k}}}_{{\rm{y}}}){\rm{f}}({{\rm{k}}}_{{\rm{x}}},{{\rm{k}}}_{{\rm{y}}}))\\  &  & \times ({\rm{rect}}(\frac{{{\rm{k}}}_{{\rm{x}}}}{2{{\rm{k}}}_{{\rm{sx}}}}){\rm{rect}}(\frac{{{\rm{k}}}_{{\rm{y}}}}{2{{\rm{k}}}_{{\rm{sy}}}})){{\rm{e}}}^{{\rm{i}}({{\rm{k}}}_{{\rm{x}}}{\rm{x}}+{{\rm{k}}}_{{\rm{y}}}{\rm{y}})}{{\rm{dk}}}_{{\rm{x}}}{{\rm{dk}}}_{{\rm{y}}}\\  & = & {{\rm{\sigma }}}_{{\rm{j}}}{{\rm{FT}}}^{-1}\{\frac{{|{\rm{f}}({{\rm{k}}}_{{\rm{x}}},{{\rm{k}}}_{{\rm{y}}})|}^{2}}{{|{\rm{f}}({{\rm{k}}}_{{\rm{x}}},{{\rm{k}}}_{{\rm{y}}})|}^{2}+\frac{{{\rm{\eta }}}^{2}{{\rm{A}}}_{{\rm{FoV}}}}{{{\rm{\sigma }}}_{{\rm{j}}}^{2}}}\}\\  &  & \ast \,\frac{1}{{{\rm{\Delta }}}^{2}}{\rm{sinc}}(\frac{{\rm{x}}}{{\rm{\Delta }}}){\rm{sinc}}(\frac{{\rm{y}}}{{\rm{\Delta }}})\end{array}$$

where * represents the convolution operator. The reconstructed current density of the point source is equivalent to the point spread function (PSF) of the system and the maximally achievable spatial resolution can be characterized by its FWHM. The first term on the right side depends on parameters of the point source (its strength, height and distance to the sensor), the area-normalized noise level and the area of the field of view. The second term depends only on the discretization parameter, i.e. the chosen in-plane resolution. Therefore, their impact on the system resolution can be analyzed separately. For example, as the pixel size decreases, the second term approaches a delta distribution and the resolution will be determined by the first term.

The first term is a low pass filter which increases in smoothness with increasing noise level. On the other hand, for large pixels, the second term dominates and determines the resolution limit of the system.

## Electronic supplementary material

Supplementary Information
