# Supplementary material for: Feasibility and resolution limits of opto-magnetic imaging of neural network activity in brain slices using color centers in diamond
Source: Sci Rep. 2018 Mar 14;8:4503. doi: 10.1038/s41598-018-22793-w (PMC5852147; doi:10.1038/s41598-018-22793-w)
Supplement: Supplementary file 1 — Supplementary Information [file 41598_2018_22793_MOESM1_ESM.pdf]

M Karadas, A M Wojciechowski, A Huck, N O Dalby, U Lund Andersen, A Thielscher

Feasibility and resolution limits of opto-magnetic imaging of neural network activity in brain slices using color centers in diamond

Supplementary Material

S1 Forward Modeling Scheme

In the following, we describe the employed forward modelling scheme to determine the extracellular electric potential from the transmembrane currents and the extracellular magnetic field from the intracellular axial currents, i.e. the current flow parallel to the cell membrane. For a cylindrical nerve structure embedded in a large extracellular medium (Fig. S1), the extracellular volume $\mathrm{V_e}$ can be approximated as an infinite homogeneous conductor. Hence, the cell membrane is the only boundary, denoted by *S*. In that case, the electric potential in the extracellular region can be written as (starting from Eqs. 26 and 27 in [1])

$$\phi_e(\mathbf{r}) = \frac{1}{4\pi\sigma_e}\int_{V_e}\frac{\nabla' \mathbf{J}_{source}(\mathbf{r}')}{|\mathbf{r}-\mathbf{r}'|}dv' + \frac{1}{4\pi\sigma_e}\int_{S}(\sigma_i\,\phi_i - \sigma_e\phi_e)\nabla'\frac{1}{|\mathbf{r}-\mathbf{r}'|}\,\mathbf{n}\,dS', \tag{S1}$$

where the operator ∇' acts on $\mathbf{r}'$. Since there are no current sources in the extracellular medium (i.e., $\nabla\,\mathbf{J}_{source}$= 0 in the extracellular space), the first term on the right hand side disappears. The extracellular potential is much smaller than the intracellular potential [2] ($\text{i.e.}, \sigma_i\phi_i \gg \sigma_e\phi_e$) and hence Eq. (S1) can be approximated as:

$$\phi_e(\mathbf{r}) = \frac{1}{4\pi\sigma_e}\int_S \sigma_i\phi_i\nabla'\frac{1}{|\mathbf{r}-\mathbf{r}'|}\,\mathbf{n}\,dS'. \tag{S2}$$

Therefore, the extracellular potential is approximately independent of the external current distribution. If the nerve structure has a small cross-section A, we can assume that ϕ_i is only changing along the structure (this direction is parameterized using the longitudinal coordinate l) and the surface integral can be written in terms of a volume integral[2]:

$$\phi_e(\mathbf{r}) = \frac{1}{4\pi\sigma_e}\int_A\left[\int_{-\infty}^{\infty}\sigma_i\frac{\partial^2\phi_i}{\partial l^2}\frac{1}{|\mathbf{r}-\mathbf{r}'|}dl\right]dA'. \tag{S3}$$

The numerical simulations of the neuron are based on core-conductor equations, which assume a constant current density across the cross-section of the cell,

$$\frac{\partial^2 \phi_i}{\partial l^2} = r_i i_m, \tag{S4}$$

where $r_i = 1/\sigma_i A$ is the intracellular resistance per unit length and i_m is the membrane current per unit length. Eq. (S4) allows us to write the extracellular potential in dependence on the membrane currents:

$$\phi_e(\mathbf{r}) = \frac{1}{4\pi\sigma_e} \int_{-\infty}^{\infty} i_m \frac{1}{|\mathbf{r}-\mathbf{r}'|} dl. \tag{S5}$$

The core-conductor model provides a good approximation as we are only interested in the transmembrane potential of the cell model[3]. Representing the nerve geometry by a series of N cylindrical compartments of length Δs_n allows discretizing Eq. (S5) as

$$\phi_e(\mathbf{r}, t) = \frac{1}{4\,\pi\sigma_e} \sum_{n=1}^{N} \int_{L_n} \frac{I_m^n(t) dl}{\Delta s_n |\mathbf{r}-\mathbf{r}_n|}\ , \tag{S6}$$

where I_m^n is the membrane current for compartment n, $\Delta s_n$is its length, and a is its radius:

$$I_m^n = i_m \Delta s_n = \frac{I_m \Delta s_n}{2\pi a}\,. \tag{S7}$$

Integrating Eq. (S6) along the center line of each compartment finally yields

$$\phi_e(\mathbf{r}, t) = \frac{1}{4\pi\sigma_e} \sum_{n=1}^{N} \frac{I_m^n(t)}{\Delta s_n} \log \left| \frac{\sqrt{h_n^2 + \rho_n^2} - h_n}{\sqrt{l_n^2 + \rho_n^2} - l_n} \right|, \tag{S8}$$

where ρ_n is the distance perpendicular to the center line of the compartment, h_n is the longitudinal distance from the end of the compartment, and $l_n = h_n + \Delta s_n$ denotes the longitudinal distance from the start of the compartment[4,5].

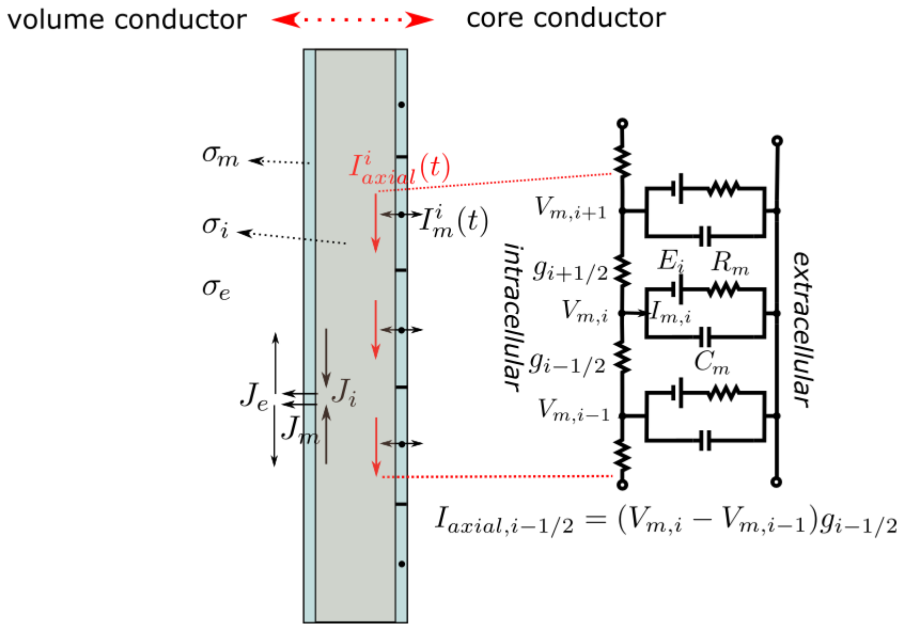

Figure S1: Model of a cylindrical neural process. The left side shows the volume-conductor model and the right side the equivalent core-conductor model. The volume conductor model distinguishes between the intracellular, membrane and extracellular regions. In the core-conductor model, the intracellular and extracellular regions are represented by axial resistances and they are connected by a membrane network[6].

A similar solution can be derived for the extracellular magnetic field (starting from Eqs. 20 and 22 in [1]) :

$$\mathbf{B}(\mathbf{r}) = \frac{\mu_0}{4\pi}\left\{\int_V \frac{\mathbf{J}_{\text{source}}(\mathbf{r}')\times(\mathbf{r}-\mathbf{r}')}{|\mathbf{r}-\mathbf{r}'|^3}\,\mathrm{dv}' - \int_V \frac{\sigma(\mathbf{r}')\nabla'\phi(\mathbf{r}')\times(\mathbf{r}-\mathbf{r}')}{|\mathbf{r}-\mathbf{r}'|^3}\,\mathrm{dv}'\right\}. \tag{S9}$$

The domain is divided into the intracellular, membrane and extracellular volumes, and the integral equations run over the complete domain. Since the extracellular and intracellular volumes are source free ohmic regions, the first integral is nonzero only at the membrane. We thus define $\mathbf{J}_{\text{source}} = \mathbf{J}_{\mathbf{m}}$ as the biochemical current sources in the membrane. The contribution of the secondary currents in the second integral of Eq. (S9) can be written for the intracellular and extracellular regions by considering the vector identities:

$$\int_{V_{i,e}} \frac{\sigma_{i,e}\nabla'\Phi_{i,e}(\mathbf{r}')\times(\mathbf{r}-\mathbf{r}')}{|\mathbf{r}-\mathbf{r}'|^3}\,\mathrm{dv}' \tag{S10}$$

$$= \int_{V_{i,e}} \frac{\nabla'\times(\sigma_{i,e}\nabla'\Phi_{i,e}(\mathbf{r}'))\times(\mathbf{r}-\mathbf{r}')}{|\mathbf{r}-\mathbf{r}'|^3}\,\mathrm{dv}'$$

$$+ \oint_{S_{i,e}} \frac{\sigma_{i,e}\nabla'\Phi_{i,e}(\mathbf{r}')\times\hat{\mathrm{n}}}{|\mathbf{r}-\mathbf{r}'|}\,\mathrm{ds}'\,.$$

For piecewise homogeneous regions, the volume integral on the right hand side is zero. Therefore, the second volume integral of Eq. (S9) can be converted into a surface integral and the magnetic field can be expressed as a sum of volume and surface integrals over the membrane [7]:

$$\mathbf{B}(\mathbf{r}) = \frac{\mu_0}{4\pi}\left\{\int_{v_m} \frac{\mathbf{J_m}(\mathbf{r}') \times (\mathbf{r}-\mathbf{r}')}{|\mathbf{r}-\mathbf{r}'|^3}dv' + \oint_{S_e} \frac{\sigma_e \nabla' \phi_e(\mathbf{r}') \times \hat{n}}{|\mathbf{r}-\mathbf{r}'|}ds' + \oint_{S_i} \frac{\sigma_i \nabla' \phi_i(\mathbf{r}') \times \hat{n}}{|\mathbf{r}-\mathbf{r}'|}ds'\right\}. \tag{S11}$$

The current densities are symmetric along the nerve structure and can be written in terms of their radial components J^r and longitudinal components J^l

$$\mathbf{J}(r,l) = J^r(r,l)\hat{\mathbf{r}} + J^l(r,l)\,\hat{\mathbf{l}} \tag{S12}$$

with $\hat{\mathbf{r}}$ and $\hat{\mathbf{l}}$ being radial and axial unit vectors, respectively. The membrane conductivity is extremely small with respect to the conductivities of the inner and outer regions. We therefore can assume that the membrane currents $\mathbf{J_m}$ have only a radial component[7]. As the membrane is very thin, the contribution of the membrane source to the magnetic field can be safely neglected and the volume integral in Eq. (S11) can be skipped.

At the inner and outer surfaces of the membrane, the radial components of the extracellular and intracellular current densities cannot contribute to the surface integrals since they are orthogonal to the surface normal $\hat{n}$. Therefore, the total magnetic field is determined by the longitudinal (axial) components of currents J_e and J_i at the outer and inner surfaces, which are defined as:

$$J_{e,i} = \sigma_{e,i}\frac{\partial \Phi_{e,i}}{\partial l} \tag{S13}$$

It can be further assumed that the external current flow $\mathbf{J_e}$ is distributed in a large volume, such that its longitudinal component at the outer membrane surface is small. In contrast, the internal current flow $\mathbf{J_i}$ is confined to a small volume and its longitudinal component is strong and dominates Eq. (S11). Thus, Eq. (S11) can be approximated as:

$$\mathbf{B}(\mathbf{r},k) \approx \frac{\mu_0}{4\pi}\oint_{S_i}\frac{J_{i,l'}}{|\mathbf{r}-\mathbf{r}'|}dl' = \frac{\mu_0}{4\pi}\int_{V_i}\frac{J_{i,l'}(\mathbf{r}') \times (\mathbf{r}-\mathbf{r}')}{|\mathbf{r}-\mathbf{r}'|^3}dv'. \tag{S14}$$

Discretizing the neuron into N compartments results in:

$$\mathbf{B}(\mathbf{r},t) = \frac{\mu_0}{4\pi}\sum_{n=1}^{N}\int_{L_n}\frac{I^n_{axial}(t)\mathbf{dl} \times (\mathbf{r}-\mathbf{r}_n)}{|\mathbf{r}-\mathbf{r}_n|^3}, \tag{S15}$$

where I^n_{axial} is the intracellular axial current for compartment n. I^n_{axial} is determined by the potential difference $V^n_m - V^{n-1}_m$ between the membrane nodes which enclose the compartment:

$$I^{n}_{axial} = J_{i,l'}\pi a^2 = \frac{V^{n}_{m} - V^{n-1}_{m}}{\Delta s_n r^{n}_{i}}. \quad (S16)$$

Integration of Eq. (S15) along the center line of each compartment finally gives

$$\mathbf{B}(\mathbf{r}, t) = \frac{\mu_0}{4\pi}\sum_{n=1}^{N}\frac{\mathbf{I}^{n}_{axial}(t)\times\hat{\rho}_n}{\rho_n}\left[\frac{h_n}{\sqrt{h_n^2 + \rho_n^2}} - \frac{l_n}{\sqrt{l_n + \rho_n^2}}\right], \quad (S17)$$

where ρ_n the distance perpendicular to the line compartment, h_n is the longitudinal distance from the end of the compartment, and $l_n = h_n - \Delta s_n$ the longitudinal distance from the start of the compartment.

S2 Comparison with the Numerical Solution for a Long Straight Axon

In the following, we validate equation (S17) by comparing the magnetic field obtained for a long straight axon with the fields calculated with the Finite-Element Method (FEM). For completeness, we also present the results obtained with two different analytical approaches, as described in [8]. The total extracellular magnetic field results from two different sources, namely the extracellular and intracellular currents. As discussed above, the magnetic field component due to the membrane currents $\mathbf{J_m}$ can be safely neglected. As first step, the distributions of the electric potential in the intra- and extracellular spaces are calculated. The potential has to satisfy Laplace's equation in the volume conductor

$$\nabla.\left(\sigma_{e,i}\nabla\phi_{e,i}\right) = 0, \quad (S18)$$

whereby we set Neumann boundary conditions at the outer and inner boundaries of the cell membrane:

$$\mathbf{n}.\left(\sigma_e\nabla\phi_e\right) = -J_n, \text{ and} \quad (S19)$$

$$\mathbf{n}.\left(\sigma_i\nabla\phi_i\right) = J_n. \quad (S20)$$

Vector **n** denotes an outwards pointing unit vector normal to the boundary. J_n represents the current density at the boundaries. For the extracellular potential, we additionally set $J_n = 0$ at the outer domain boundary. For the cell membrane, J_n is identical to the (dominant) radial component of the membrane current density $\mathbf{J_m}$. For the core-conductor model, J_n can thus be determined from the membrane current per compartment

$$J_n = \frac{I^{n}_{m}}{\Delta s_n 2\pi a}, \quad (S21)$$

with Δs_n being the length and a the radius of compartment n. After determining ϕ_e and ϕ_i, the current densities are calculated using Ohm's law $\mathbf{J} = \sigma_{e,i}\nabla\phi_{e,i}$, and the Biot-Savart law is used to determine the contributions of the external and internal current flows to the total extracellular magnetic field.

The modelled geometry is depicted in Fig. S2A&B. The membrane potential and membrane currents were simulated using NEURON, and the results fed into the FEM calculations based on COMSOL Multiphysics (v5.3). We made use of the axisymmetric geometry around the Y axis to set up a 2D triangle mesh with a symmetry constraint at z=0 in order to describe the geometry in a computationally efficient way. The geometry corresponds to a cylinder of 40 mm height (in Y direction) and 60 mm diameter (corresponding to 30 mm in Z direction), with the nerve being placed in its center. The triangle density in the region close to the nerve membrane was strongly increased to account for the thin diameter of the axon. Quadratic finite elements were employed, and the relative tolerance for the conjugate gradient solver were required to be $<10^{-3}$.

The axon has active Hodgkin-Huxley channel dynamics and a length of 3.2 mm. It is discretized in compartments with lengths smaller than the electrotonic space constant. Its diameter was chosen small (5 µm) in order to be in the range seen for hippocampus cells. The following parameters were used:

radius a	thickness t_m	internal conductivity σ_i	membrane conductivity σ_m
$2.5\ \mu m$	$13.7\ nm$	$1\ \Omega^{-1}m^{-1}$	$10^{-5}\ \Omega^{-1}m^{-1}$
length L	velocity u	external conductivity σ_e	membrane permittivity ϵ_m
$3.2\ mm$	$2\ ms^{-1}$	$0.3\ \Omega^{-1}m^{-1}$	$6.195 \times 10^{-12} C^2 N^{-1} m^2$

The results were evaluated at an (arbitrarily chosen) time point when AP had traveled 2/3 of the length of the axon. Fig. S2C shows the transmembrane potential along the axon. The magnetic field determined by our forward modelling scheme (Eq. (S17); abbr. **BS** – black curve in Fig. S2D) was compared with the total magnetic field obtained via the FEM simulations (abbr. **FEM** – blue curve in Fig. S2D), showing a very good agreement. From this, we can conclude that our forward modeling scheme based on Eq. (S17) is accurate when the assumption of a large homogeneous extracellular volume is valid.

In addition, the results obtained with two different analytical approaches, as described in [8], are shown. Those approaches allow calculating the magnetic field from the simulated membrane potential, based on the representation of the axon as an infinite cylinder of radius a embedded in an isotropic unbounded outer region. The first analytical method, based on Ampere's law (**B Ampere** – purple curve in Fig. S2D), strongly overestimates the magnetic field caused by the internal current flow when the distance to the nerve is large, which is in accordance to the known limitations of this approach [8]. The second method that evaluates the Biot-Savart law in the spatial frequency domain (**BS kspace** – red dashed curve in Fig. S2D) performs better, but systematically underestimates the field strength at all distances. This effects only occurs for thin axon radii as tested here, while it is accurate for thicker axons as simulated in the original study [8].

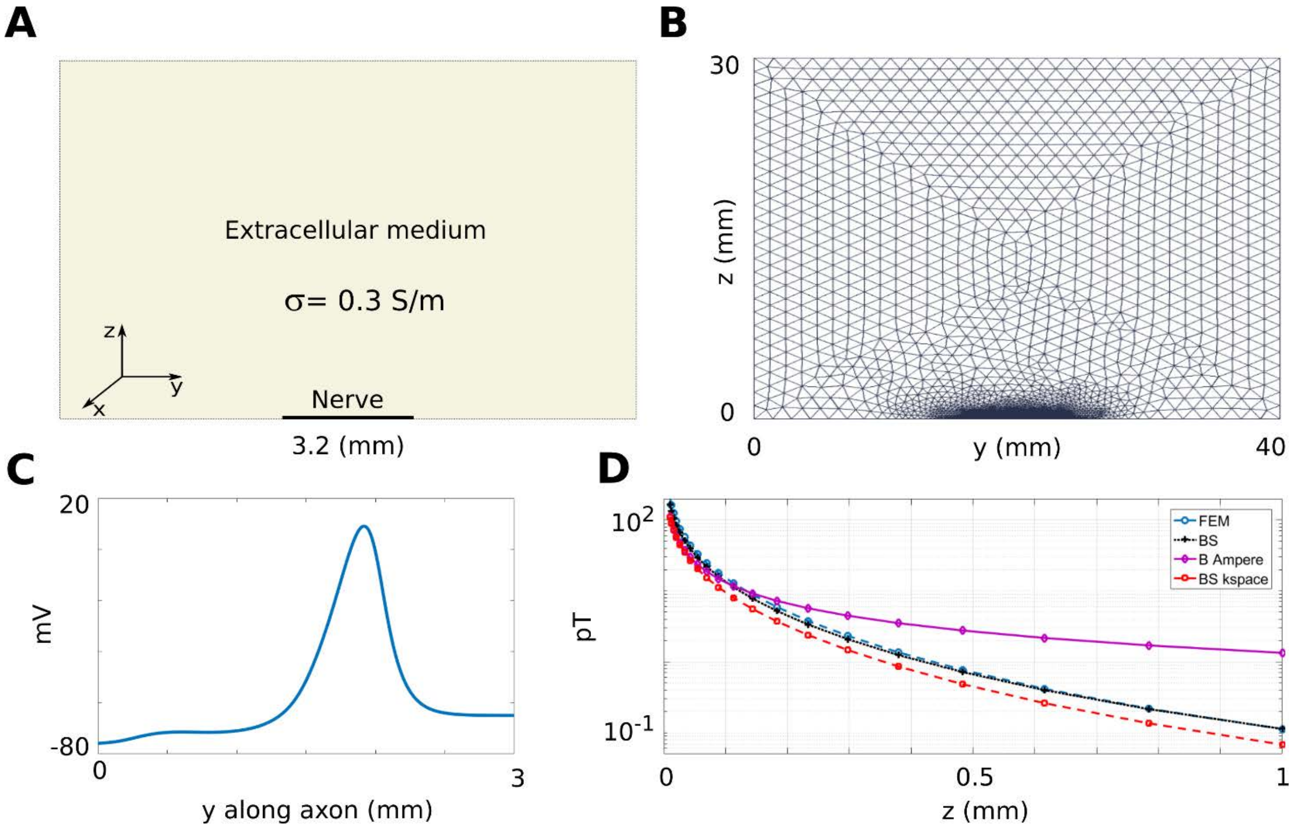

Figure S2: (A) Schematic image of the simulated straight axon, modelled in 2D as axisymmetric geometry around the Y axis (for better visibility, the length of the axon is not drawn to scale). **(B)** Visualization of the triangular mesh used to represent the geometry. The triangular density is increased close to the nerve in order to ensure an accurate solution. **(C)** Transmembrane potential along the axon, plotted at an arbitrarily chosen time point when the AP had traveled 2/3 of the length of the axon. **(D)** Comparison of analytically calculated and numerically simulated magnetic fields of the straight axon. Plotted are peak-to-peak amplitudes of the magnetic field strength determined at y = 1.8 mm, corresponding to the strongest slope of the membrane depolarization and thus the peak position of the magnetic field, in dependence on the distance to the nerve. The four modelling schemes are: Finite element method (**FEM**), accounting for the total magnetic field of the internal and external current flow; Core-conductor model of the axon combined with the Biot-Savart law to estimate the magnetic field of the internal current flow (**BS**); Analytical solution for the magnetic field caused by the internal currents flow in an infinite straight axon, based on the Biot-Savart law in the spatial frequency domain (**BS kspace**); Analytical solution for the magnetic field caused by the internal current flow in an infinite straight axon, based on Ampere's law (**B Ampere**).

S3 Correction for the effects of tissue anisotropy and boundaries

The forward modeling scheme so far assumed that the extracellular volume V_e can be well approximated as an infinite homogeneous conductor. However, the brain slice will be embedded in a saline bath, resulting in a conductivity boundary between brain and saline. In addition, an anisotropic electrical conductivity has been reported, e.g. for the guinea-pig hippocampus [9]. Finally, also the boundaries between the slice/saline and the

surrounding non-conductive air, plastic and diamond affects the extracellular current pathways. The effect of conductivity discontinuities on electric potential recording was explored for Multielectrode array in detail [10]. In the following, we investigate the impact of these conductivity discontinuities on the magnetic field created by a straight nerve fiber. For this, we employ numerical simulations based on the finite element method (FEM) to determine the magnetic field contribution by the extracellular currents (Eqs. S18 to S21), while determining the contribution of the internal currents directly from Eq. (S17). This allows us to derive multiplicative factors that can be applied to the results of the forward modeling scheme to approximately correct for the impact of the finite extracellular volume. We apply these factors to rescale the magnetic fields of the pyramidal neurons investigated in the main part of the study in dependence of their distance to the diamond surface. While the large number and the histological complexity of the simulated pyramidal neurons prevents a direct assessment of their extracellular currents via FEM, our strategy allows us to account for the major effects caused by the extracellular volume.

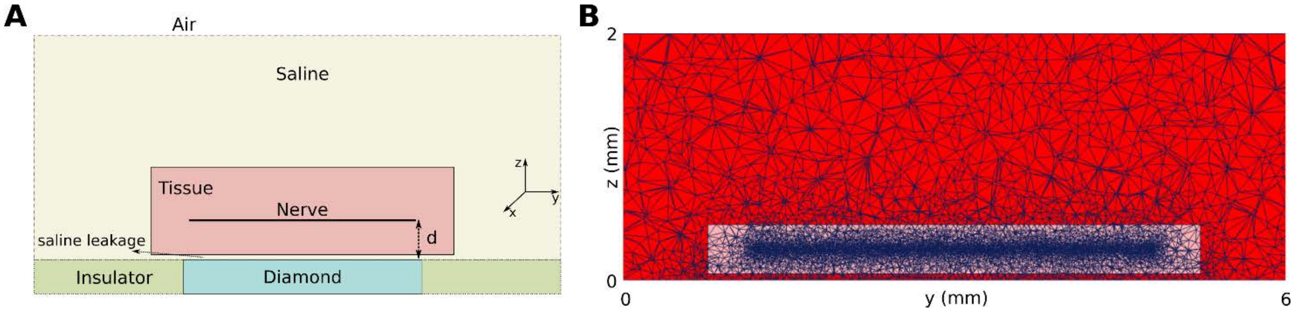

Figure S3: (A) Schematic image of the simulated straight axon embedded in brain tissue and surrounded by saline. **(B)** Visualization of the tetrahedral mesh used to represent the geometry. The tetrahedral density is increased close to the nerve in order to ensure an accurate solution.

The modelled geometry is depicted in Fig. S3A. The hippocampus slice has a volume of 1x4x0.4 mm^3, and the outer dimensions of the saline layer are 2x6x2 mm^3. A single nerve with 5 µm diameter and 3.2 mm length is placed centrally within the hippocampus slice at a distance d from the diamond surface. The following four scenarios were simulated: 1) Isotropic extracellular space with a conductivity of $\sigma_e = 0.3$ S/m, without distinction between saline and hippocampal tissue (**scenario NS**). 2) Saline with a conductivity of $\sigma_S = 1.5$ S/m and isotropic tissue with a conductivity of $\sigma_T = 0.3$ S/m (**scenario S**) [11]. 3) Saline with anisotropic hippocampal tissue (**scenario S+A**). The tissue conductivity along the nerve cell was set to $\sigma_{Ty} = 0.45$ S/m, while the conductivity normal to the nerve was kept at $\sigma_T = 0.3$ S/m. 4) Saline with anisotropic hippocampal tissue and an additional leakage layer of 20 µm height between the tissue sample and the diamond, filled with saline (**scenario S+A+L**). In order to test how the magnetic field caused by the extracellular current flow depends on the distance of the nerve to the diamond, the latter was varied from 50 to 300 µm in 50 µm steps. The membrane currents were calculated in NEURON, employing the same cell dynamics as in the

previous paragraph. FEM calculations based on COMSOL Multiphysics (v5.3) were then used to determine the extracellular current density and the resulting magnetic field. The extracellular region was modelled as tetrahedral mesh, with an increased tetrahedral density close to the nerve fiber. Quadratic finite elements were employed, and the relative tolerance for the conjugate gradient solver were required to be $<10^{-3}$.

Since the nerve is aligned along the Y direction, only the B_X and B_Z components of the magnetic field are strong at the diamond surface and are analyzed further. Figure S4 shows snapshots of the B_X component caused by the external current flow at the surface, sampled along a line in Y direction underneath the axon at an (arbitrarily chosen) time point at which the AP had traveled 2/3 of the length of the axon. All four scenarios show similar results, indicating that the outer boundaries of the volume conductor have the strongest impact, while the other modelled features play only a minor role (please compare to the results for the infinite extracellular volume shown in Fig. S2C). Figure S5 shows the parts of the B_X component caused by the internal and external current flow for the A+S scenario, revealing that the impact of the external currents is increased by around three orders of magnitude compared to an infinite extracellular volume. The nerve is far closer to the diamond surface than to the upper boundary of the saline bath, so that the major contribution comes from the diamond surface, which causes an increased current density in the extracellular space between the nerve cell and diamond. Interestingly, this effect gets stronger with increasing distance between the nerve and the surface, as this increases the overall amount of current in the space between the nerve cell and diamond. Figure S6 shows the corresponding snapshots for the B_Z component at the surface, sampled along a line in X direction. While the part of the field caused by the external current flow is still increased compared to the case of an infinite extracellular volume, this effect is far weaker compared to the B_X component and can be ignored in first approximation.

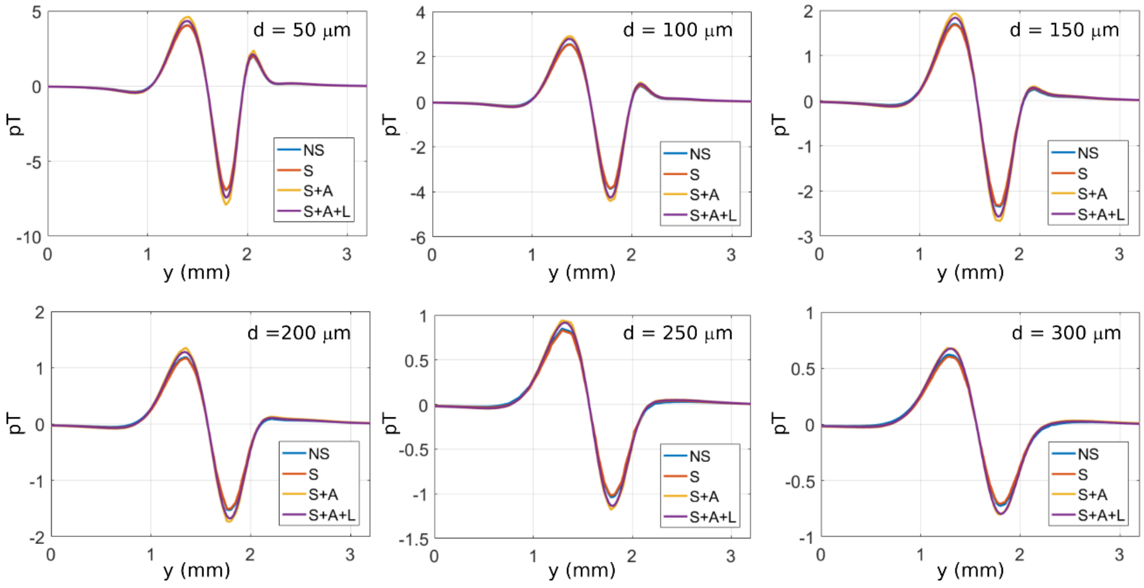

Figure S4: Impact of inhomogeneous and anisotropic conductivities of the finite extracellular volume conductor shown in Fig. S3 on the magnetic field. Shown is the B_X component caused by the external current flow, sampled at the diamond surface along a line in Y direction underneath the axon. The successive plots show the results for increasing distance of the nerve to the diamond. The four scenarios give similar results.

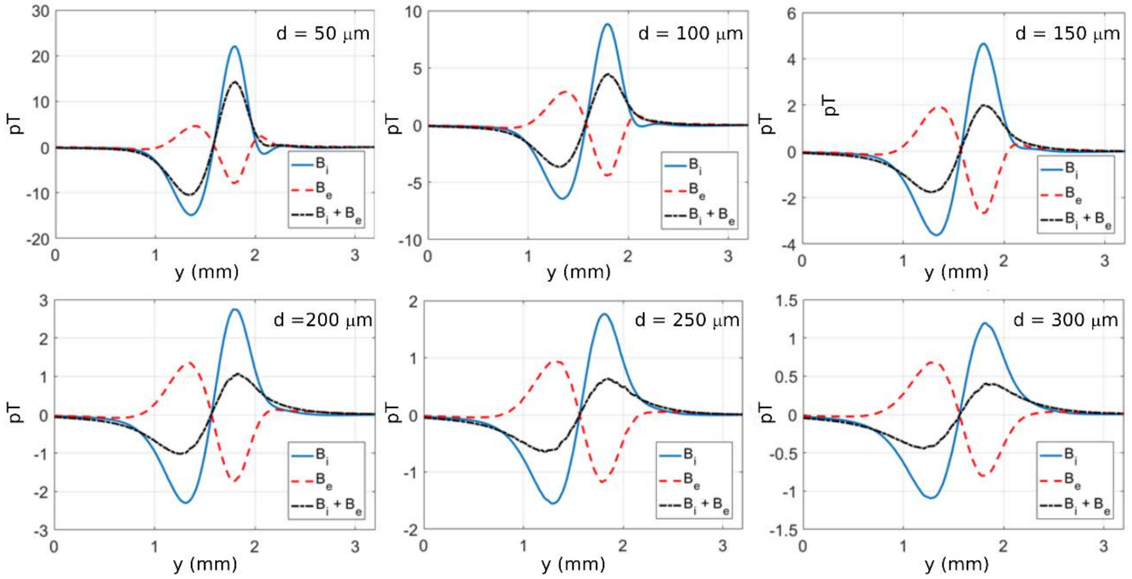

Figure S5: B_X components of the magnetic field caused by the internal and external current flow (denoted as $B_{X,I}$ and $B_{X,E}$) for the S+A scenario. The field was sampled at the diamond surface along a line in Y direction underneath the axon. The successive plots show the results for increasing distance of the nerve to the diamond.

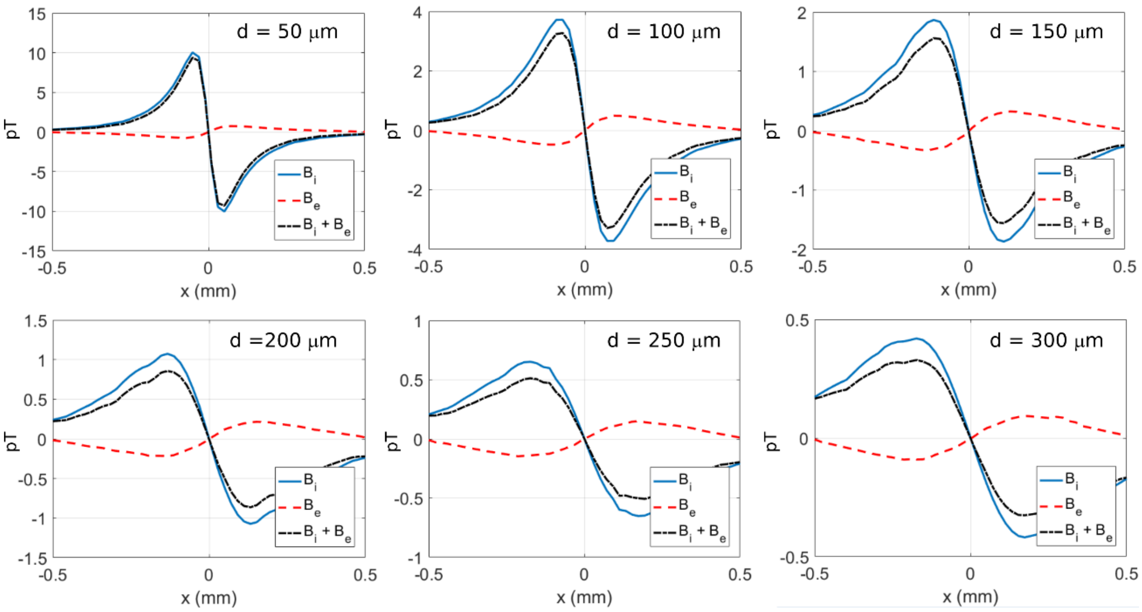

Figure S6: B_Z components of the magnetic field caused by the internal and external current flow (denoted as $B_{Z,I}$ and $B_{Z,E}$) for the S+A scenario. The field was sampled at the diamond surface along a line in X direction underneath the axon. The Y coordinate of the line was chosen according to the peak of B_Z.

We were further interested how the spatial distribution of B_X and B_Z at the diamond surface is affected by the finite extracellular volume conductor. Figure S7 exemplarily shows the results for a nerve placed at a distance of 150 µm from sensor plane for the S+A scenario. As expected, the spatial distribution of B_Z is hardly affected by the external current flow. In contrast, B_X gets "narrower" in X direction, while mostly keeping its shape in Y direction.

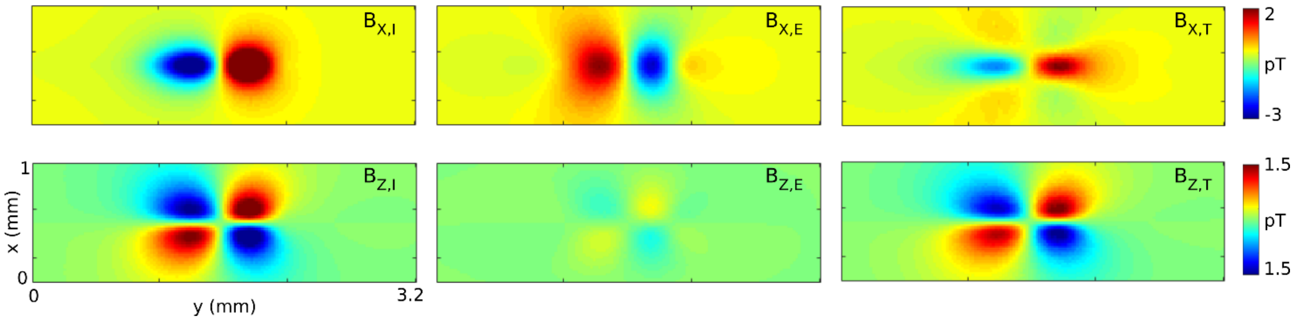

Figure S7: Spatial distribution of B_X and B_Z on the diamond surface for a nerve at a distance of 150 µm (S+A scenario). Shown are the parts caused by the internal (left column) and external (middle column) current flow, and the total field (right column).

Finally, we determined distance-dependent scale factors to correct the results obtained with the simplified forward modeling scheme used for the pyramidal neurons in the main part of the paper (Fig. S8A). The scale factors are the ratio between the total field component and the part due to the internal current flow, calculated at the position where the field component peaks. While B_Z requires hardly any rescaling, B_X is reduced by up to 60%. For B_X, an analytical expression for the scale factor is derived for use in the sensitivity analysis. For that, a rational polynomial is fitted to the data in dependence of the distance d (in µm), resulting in $s(d) = 0.25 + \frac{42.6}{d+52}$. For B_Z, the coefficient of determination between the magnetic field distributions of the internal and total current flow are close to 1 for all simulated distances between nerve and diamond surface (Fig. S8B). The impact of the finite extracellular volume conductor is larger for the spatial distribution of B_X, with the coefficient of determination varying between 0.83 and 0.94. As visible in Fig. S7, this is mainly due to a narrowing of B_X in the direction normal to the nerve fiber. This effect is neglected in the results shown for the pyramidal cells in the main part of the paper, which were only corrected using the scale factors. As a consequence, the magnetic fields will be spatially slightly more confined than shown there, and also the achievable spatial resolution will be slightly underestimated.

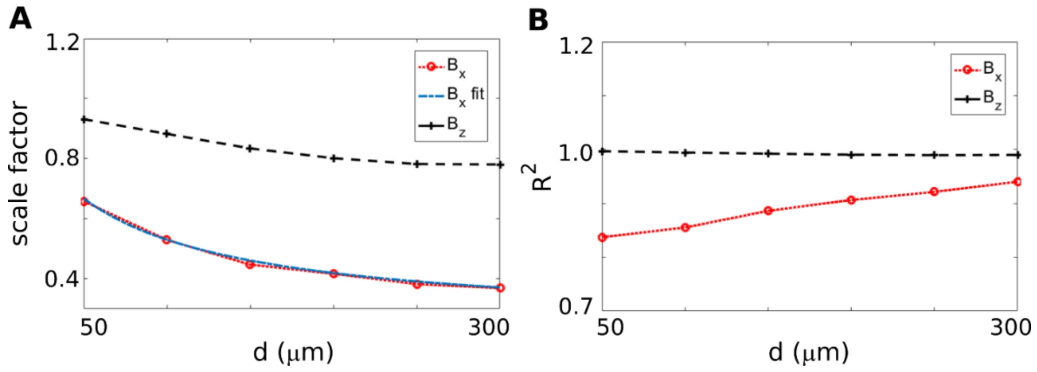

Figure S8: Effect of the finite extracellular volume conductor (S+A scenario) on the strength and spatial distribution of B_X and B_Z. **(A)** Scale factors (ratios between the total field component and the part caused by the internal current flow, i.e. $B_{X,T}/B_{X,I}$ and $B_{Z,T}/B_{Z,I}$) in dependence of the distance to the diamond surface. The factors were determined at the peak positions of B_X and B_Z, respectively, on the diamond surface. The blue line (B_{X_fit}) depicts the polynomial $s(d) = 0.25 + \frac{42.6}{d+52}$ fitted to B_X. **(B)** Coefficient of determination between the spatial distributions of the total field components ($B_{X,T}$ and $B_{Z,T}$) and the parts caused by the internal current flow ($B_{X,I}$ and $B_{Z,I}$) on the diamond surface. The results were determined in dependence of the distance to the surface.

For comparison, we also tested the impact of the finite extracellular volume conductor on the electric potential at the diamond surface (Fig. S9). Interestingly, the electric potential depends more strongly on the volume conductor properties than the magnetic field. The tendency is that stronger electric potentials occur at the surface compared to the infinite homogenous volume conductor (denoted NB in Fig. S9A). However, this effect disappears when a thin saline "leakage" layer between the tissue compartment and the surface is introduced (scenario S+A+L). The relatively well conductive saline layer strongly reduces the potential differences at the surface. A similar result is seen for the spatial distribution of the electric potential at the surface (Fig. S9B), with the NB and S+A+L scenarios giving very similar results. Considering this similarity, we decided against applying correction factors for the electric potential in the main part of the study.

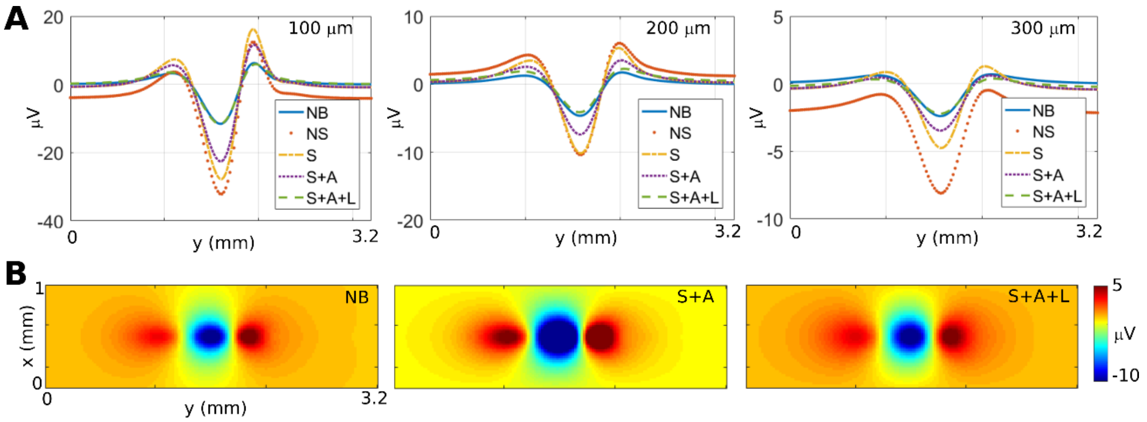

Figure S9: Effect of the finite extracellular volume conductor on the strength and spatial distribution of the *electric potential* at the diamond surface. **(A)** Electric potential at the diamond surface, sampled along a line in Y direction underneath the axon, for three different distances between the axon and the surface. The same four scenarios were tested as for the magnetic field. In addition, the results for an infinite homogeneous extracellular volume conductor are shown for comparison (termed NB - no boundaries). **(B)** Spatial distribution of the electric potential for a nerve placed at 200 µm distance from the diamond surface. When a thin saline "leakage" layer is introduced between the tissue and the diamond surface (scenario S+A+L), the field is almost identical to that obtained for an infinite homogeneous extracellular volume conductor.

S4 Comparison with Existing Studies: Single Axon case

We modeled a single axon to compare it with previously reported magnetometry measurements of axonal action potentials [12]. In that study, NV magnetometry measurements were performed for excised giant axons of two species, namely *Myxicola infundibulum* (worm) and *Loligo pealeii* (squid). In addition, recordings from axonal activity in intact worms were performed. Here, we compare our simulations based on the forward modeling scheme (Eq. S17) with their results for the excised worm axon and the intact worm. In their first experiment, the giant axon of the worm was excised and measured at a temperature of 21°C and a standoff distance of 0.3 mm to the nerve center. In their second experiment, the worm was kept at 10 °C and the measurements were taken at a distance 1.2 mm from the nerve center. To compare our numerical methods with the reported measurement results, we modeled single axons with $\sigma_i = 1.5/\Omega m$ and diameters ranging between 200 µm and 400 µm in NEURON and estimated the resulting magnetic field based on the forward modeling scheme outlined above. The assumed temperatures were either 21°C (excised axon) or 10°C (axon of living worm). In the excised axon (Fig. S10A), the simulated peak magnetic field strength increases from around 1 to 3.5 nT with increasing diameter, which overlaps well with the measured field strengths. For the axonal activity of the living worm (Fig. S10B), the peak magnetic field strength decreases to one fourth of the one observed for the excised axon due to the increased distance. In addition, its duration is longer due to the lower ambient temperature that slows down the action potential. Our simulation results are in good agreement with the previously presented measurements [12], which show that the peak field strength was around 0.4 nT for a diameter of 300 µm. It should be noted that we did not correct for the magnetic field of currents in the extracellular space, as the correction factors derived above are specific for the geometry of the envisioned recordings from the hippocampus slices.

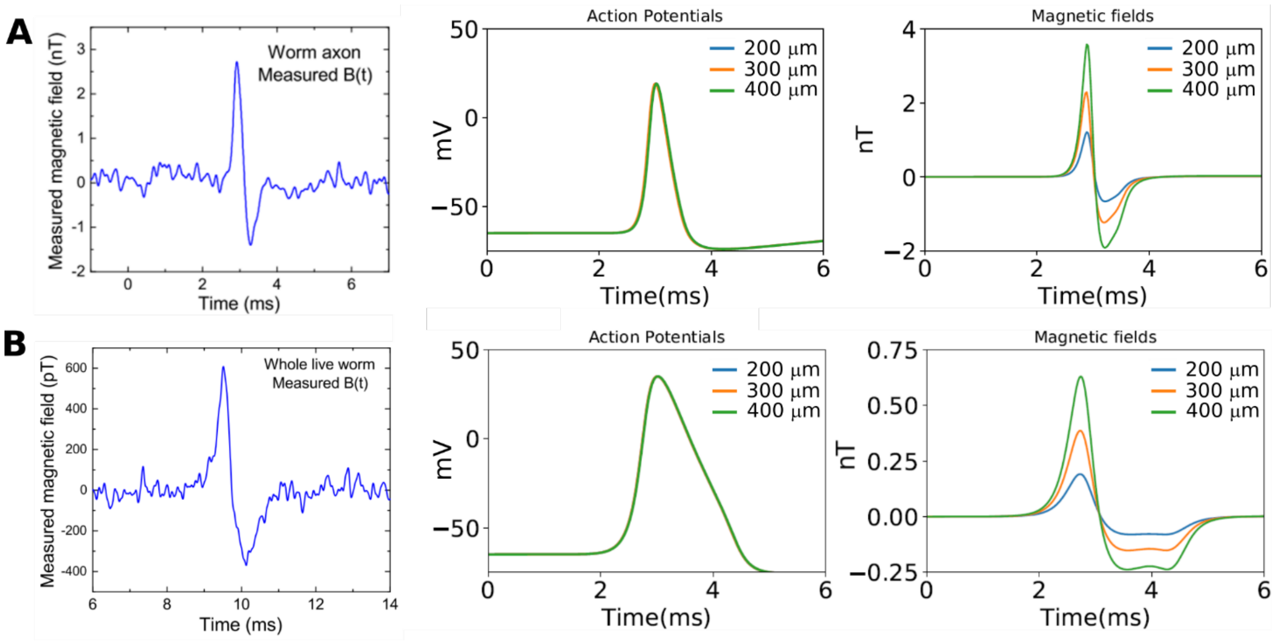

Figure S10: Single point measurement and simulations of the magnetic field. **(A)** Excised single giant axon at 21°C. Left: Measured magnetic field at distance of 300 µm from the center of the nerve (reproduced from [12]). Middle: Simulated action potential for different axon diameter. Right: Calculated total magnetic field at a distance of 300 µm. **(B)** Axonal activity of a living worm at 10 °C. Left: Measured magnetic field at distance of 1.2 mm from center of nerve (reproduced from [12]). Middle: Simulated action potential for different axon diameters. Right: Calculated total magnetic field at distance of 1.2 mm.

S5 Neural cell dynamics of the CA1 pyramidal cells

The CA1 pyramidal cells of the third scenario are modelled using previously reported morphology and biophysical properties [13]. The model files are publically available under the ModelDB section of the Senselab database (http://senselab.med.yale.edu). The passive elements of the model comprise the cell membrane with a capacitance of $C_m = 1\ \mu F/cm$ and a resistance of $R_m = 28\ k\Omega cm^2$, and the intracellular space with a resistance of $R_a = 150\ \Omega cm$. The active model elements comprise voltage-gated sodium channels and different potassium membrane currents (A-type and delayed rectifiers), with further details outlined next. Electrical stimulation of the Schaffer collaterals results in a temporally synchronous synaptic excitation of the CA1 pyramidal cells that are targeted by the collaterals. Here, this is simulated by excitation of the AMPA synapses of the pyramidal cells, which are situated on the apical dendrites (the stratum radiatum, S.R.) and the basal dendrites (the stratum oriens, S.O.). The synaptic events are modelled by time-dependent changes of the conductance of the synapses. These conductance changes are described by a double-exponential function $g_{syn} = \bar{g}_{syn} A^{-1}\left[e^{-t/\tau_{rise}} - e^{-t/\tau_{decay}}\right]$, with constant $\tau_{rise} = 1.5\ ms$ controlling the rise time, constant $\tau_{decay} = 2.5\ ms$ controlling the decay time and $\bar{g}_{syn}$ denoting the peak conductance[14]. Constant A

is used for normalization to set the maximum value of g_{syn} to be $\overline{g}_{syn}$. These synaptic events inject currents of $I_{syn} = g_{syn}(V_m - V_{rev})$ at the membrane positions of the synapses (a reverse potential of $V_{rev} = 0\ \mathrm{mV}$ is used). The simulations of the neural dynamics are conducted in NEURON, using the backward-Euler integration method to solve the cable equation with a fixed time step of 25 µs. For that, each section of a CA1 pyramidal cell (such as a dendritic branch) is subdivided into multiple cylindrical compartments to achieve compartment lengths smaller than the electrotonic space constant λ. Furthermore, each section is forced to have at least three compartments to be able to calculate axial currents from the membrane voltages as input to the above forward modelling scheme. The resulting models of CA1 pyramidal cells consist of 1427 compartments.

The stimulation of a CA1 pyramidal cell via the Schaffer collaterals is mimicked by synaptic events, which are generated at 40 randomly selected locations at the s. oriens and 40 random positions at the s. radiatum[15]. The time points of the synaptic events are modelled by truncated random functions with a normal distribution as follows:

1. Generate an excitation time T_{exc}
$$T_{exc} = \left(\frac{T_{start} + T_{stop}}{2}\right) + \left(\frac{T_{stop} - T_{start}}{4}\right)\mathcal{N}(0, \sigma)$$
2. If $T_{start} \leq T_{exc} \leq T_{stop}$, assign T_{exc} as time point of the synaptic event, otherwise regenerate T_{exc} until it remains in the desired time window.

$\mathcal{N}(0, \sigma)$ is a normal distribution with zero mean and standard deviation σ ($\sigma = 0.25$, unless indicated differently; corresponding to a standard deviation of 1.56 expressed in ms), and represents the time jitter in the excitatory inputs. The first wave of synaptic events is generated using $T_{start} = 0$ and $T_{stop} = 25\ \mathrm{ms}$, and a second wave is generated using $T_{start} = 25$ and $T_{stop} = 50\ \mathrm{ms}$ in order to mimic repeated electric stimulation at 40 Hz. The synaptic strength is controlled by setting the peak conductance $\overline{g}_{syn}$. In order to evaluate an upper limit for the magnetic field strengths that can occur merely due to EPSPs without inducing action potentials, a value of 0.3 nS was chosen. Consistent spiking activity could be created by selecting a strength of 0.6 nS (or higher, if indicated). It is worth noting that the number of modelled excitatory synaptic inputs is far lower than occurring for real neurons. In order to reduce the computational complexity of the simulations, the effects of a high number of excitatory synapses is mimicked by increasing the synaptic strengths instead.

S6 Comparison with Existing Studies: Recordings from the hippocampus CA1 subfield with μ-SQUIDs

We modeled a hippocampus slice to compare it with previously reported magnetometry measurements from the hippocampus CA1 subfield of the guinea-pig based on a μ-SQUIDs and small pickup coils [16]. In that study, a longitudinal hippocampus slice with 5 x 2 x 0.4 mm was used. The component of the magnetic field normal to the slice surface was recorded using μ-SQUIDs connected to coils with 4 mm diameter. The coils had a distance of 2 mm to the upper surface of the slice. Given the size of the pickup coils and the distance to the slice, their achievable spatial resolution is far lower than that of our envisioned approach. However, in our context, their measurement data is useful to further validate our modeling approach. For that, we simulated the topography of the neural magnetic fields and compared the simulation results with the recorded topographies reported in [16]. We modeled a longitudinal CA1 slice with 5 x 2 x 0.4 mm. Electrical stimulation of the Schaffer collaterals was simulated by activation of the AMPA synapses to generate spiking events across the complete slice. The component of the neural magnetic field normal to the slice was calculated, and its topography was determined in an area of 20 mm x 12 mm centered above the slice at a distance of 4 mm. The area was divided into 15 regions, and the average of the normal field component was determined in each of the regions. The experimental study reported peak fields of 15 pT for population spikes, which is in the same order of magnitude but lower (Fig.S11). There are several possible reasons for the remaining differences: First, the number of activated cells could have been less in the experimental study, or the simulated cell density could have been different to the density in the measured guinea-pig brain slices. Second, the neural event types causing activation are different. Consistent with the main scenario modeled in our study, we simulated neural excitation via the stimulation of Schaffer collaterals, resulting in highly coherent activation of the pyramidal cells. In contrast, the experimental study used direct electrical stimulation of the CA1 cells with 6 electrodes distributed across the CA1 subarea. For the latter case, a lower synchrony between the activated pyramidal neurons could have reduced the magnetic field peak of the population spike. Finally, we did not account for the finite extracellular volume conductor in our simulations. However, we do not expect a too strong influence in this case, as the magnetic field component normal to the boundary between conductive tissue and non-conductive material (the diamond and dewar, respectively) is hardly affected (see results for B_Z in Fig. S8).

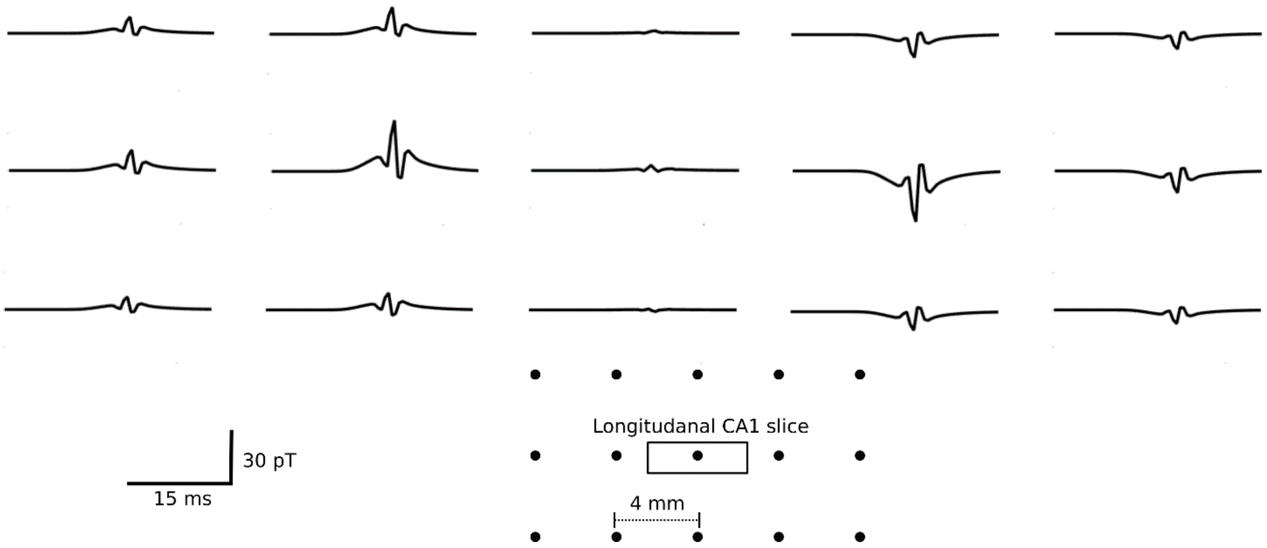

Figure S11: Topography of the simulated neural magnetic fields due to activation of a longitudinal CA1 slice, recorded by µ-SQUIDs. The centers of the pickup coils were placed on a regular grid with a 4 mm spacing (as indicated by the black dots). The brain slice is indicated by the rectangle. Shown is the magnetic field component normal to the slice. The field topography (strong directly neighboring the slice, with opposite signs for left and right positions; weak directly above the slice) shows that the magnetic fields are generated by longitudinal currents flowing along the cell bodies.

S7 Computation of the True Volume Current Density

During the computations of the neural magnetic fields, the segments of neurons are modelled as current-carrying wires. However, during the reconstruction of the current density from the simulated magnetic field measurements, the currents are assumed to be volume currents. In the following, we therefore derive a mapping between the axial currents and the corresponding volume current densities for comparison with the reconstructed current densities, y. In our modeling, we computed the total current within a voxel by integrating the axial current over the neuron segments in this voxel. The volume current density is then estimated by dividing by the volume of voxel

$$\mathbf{J}_\mathrm{i} = \frac{1}{\mathrm{V_i}} \sum_{\mathrm{for\ n\ inside\ i}} \mathrm{I}^{\mathrm{n}}_{\mathrm{axial}} \mathbf{L}^{\mathrm{n}}_{\mathrm{i}}, \tag{S22}$$

where V_i is the volume of voxel and $\mathrm{L}_\mathrm{i}^\mathrm{n}$ is the xy projection of segment vector n to voxel i.

For the CA1 slice, the volume current density was computed at 64x64 points with a total area of 1 mm^2 and an assumed thickness of 0.3 mm. For the planar cell, the voxel dimension was chosen to be 4x4x2 μm^3 for a total area of 0.09 mm^2. To test the validity of our estimation, the magnetic fields of both the CA1 slice and the planar cell were determined from the volume current densities using the frequency domain approach described in [8] and compared with the magnetic field determined from the axial currents (Fig. S12). The

simulation parameters were kept same as for Fig. 2 and Fig. 3 of the main text. The volume currents, projected onto the measurement plane of the sensor, give consistent results compared to the actual axial sources, except small discrepancies that originate from discretization errors. Moreover, in the planar cell model, a thickness of 2 µm is chosen based on the mean radius of segments, resulting in higher fields around the thicker soma layer.

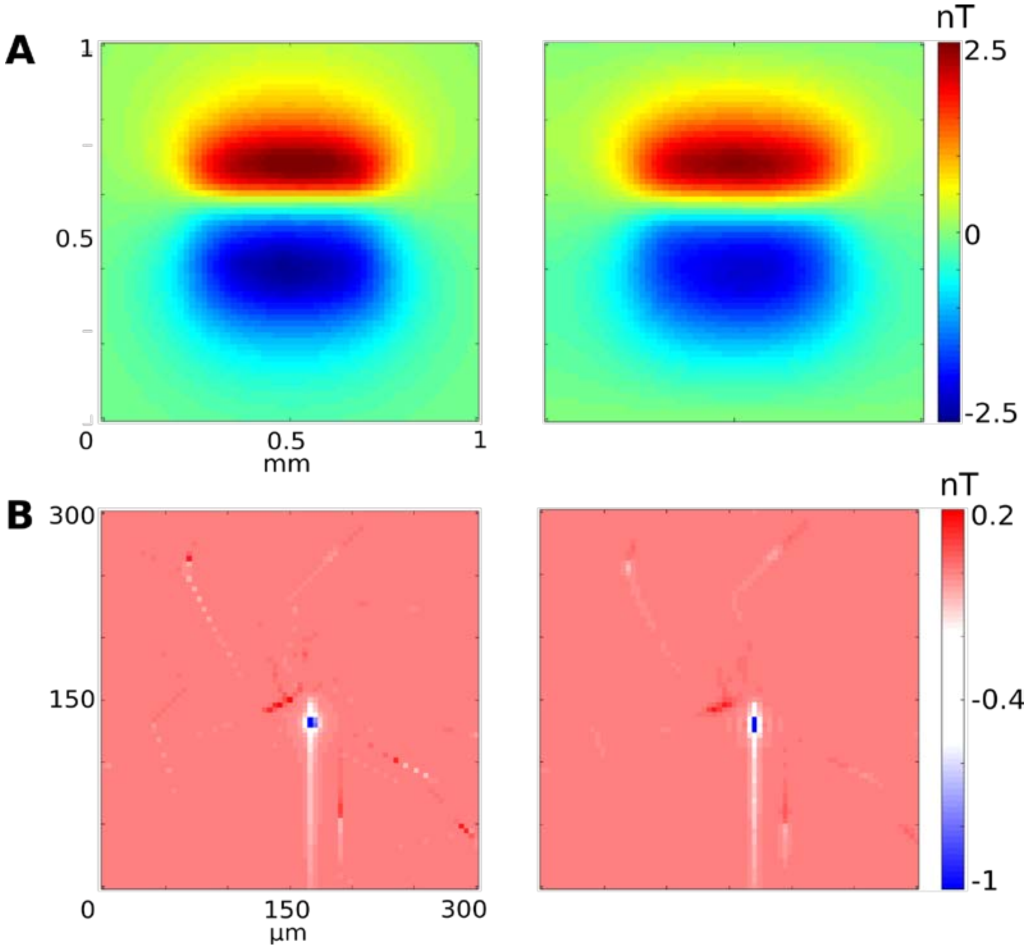

Figure S12: Simulated magnetic field of the CA1 slice and the planar cell. **(A)** The magnetic field of the CA1 subarea, determined from the axial currents (left) vs projected volume currents (right). (B) The magnetic field of the planar cell computed from the axial currents vs projected volume currents.

Additional Supplementary Figures

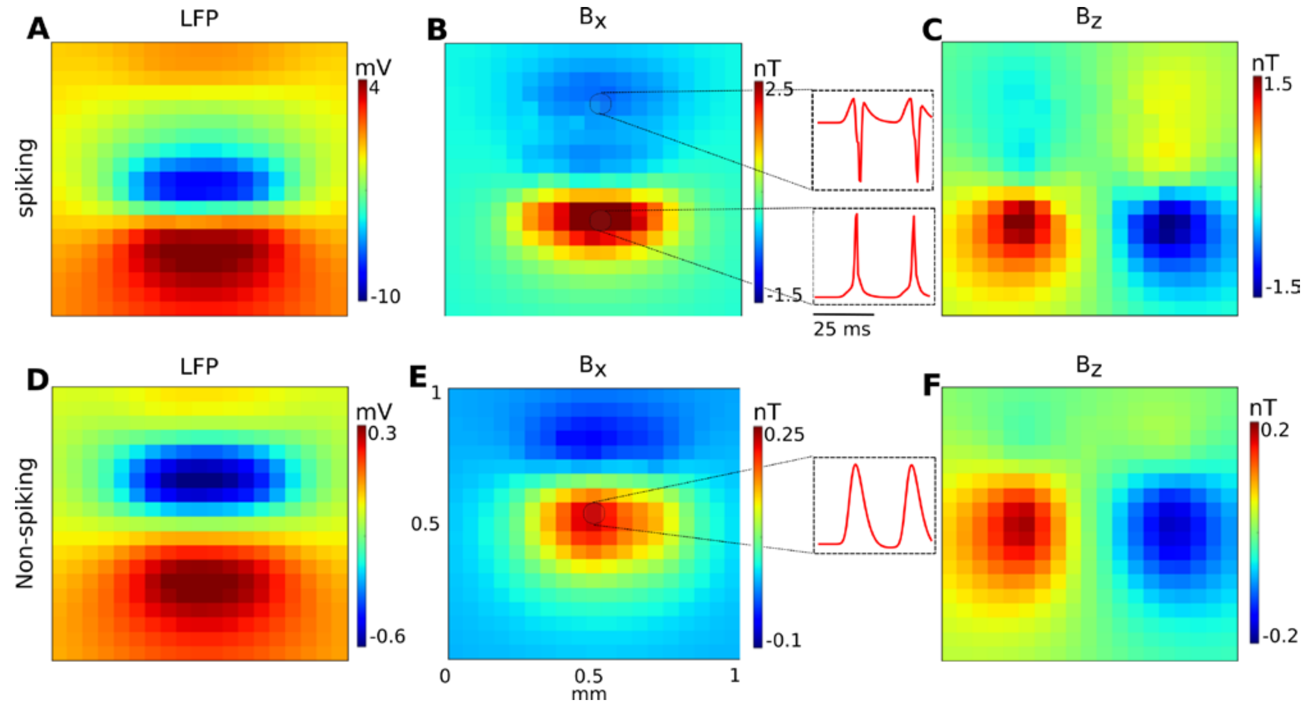

Figure S13: Simulation results showing the spatial distribution and the temporal shape of the extracellular fields for the CA1 subarea for the case that synaptic activity only occurs at the S.R. (**A**) Local field potential (LFP) for the **spiking** case. (**B**) B_X component of the magnetic field (spiking case). (**C**) B_Z component of the magnetic field (spiking case). The **insets** depict the temporal shapes of the magnetic field components, extracted from the indicated positions (a time window of 50 ms is shown). The initial phases before the action potentials reflect the accumulation of excitatory postsynaptic potentials (EPSPs). (**D**) LFP for the **non-spiking** case. (**E**) B_X component of the magnetic field (non-spiking case). (**F**) B_Z component of the magnetic field (non-spiking case). The temporal shapes depicted in the insets reflects the accumulation of EPSPs.

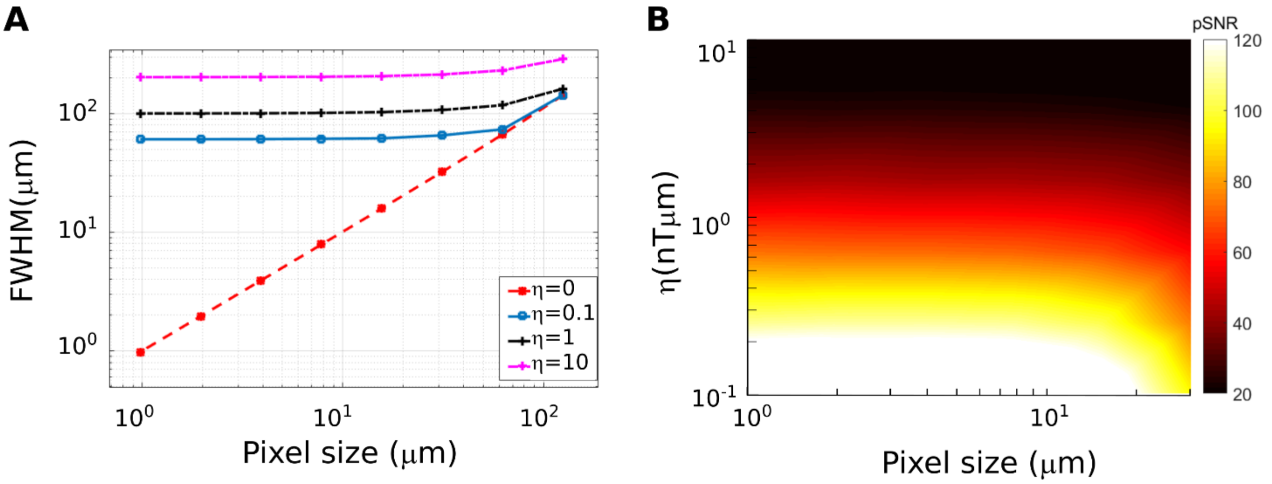

Figure S14: Recording from the CA1 subarea. This figure complements Fig. 7 in the main paper. Here, the neural source has a height of 50 μm (instead of 300 μm) in Z direction, starting at a distance of 50 μm from the diamond surface. Correspondingly, the total current induced by the source in Y direction is 1/6 of the current for the source in Fig. 7. **A)** Shown is the achievable system resolution (characterized by the full width at half maximum – FWHM – of the reconstructed neural axial current density) in dependence on the in-plane resolution of the sensor. The achieved system resolution is almost constant for higher in-plane resolutions, but drops off quickly for too low in-plane resolutions. **(B)** Peak signal-to-noise ratio (pSNR) for recordings from the CA1 subarea as a function of the in-plane resolution of the sensor (pixel size) and the signal-to-noise power of the source, η. As long as the in-plane resolution is chosen high enough to keep the best possible system resolution, also the pSNR of the reconstructed axial current densities remains constant.

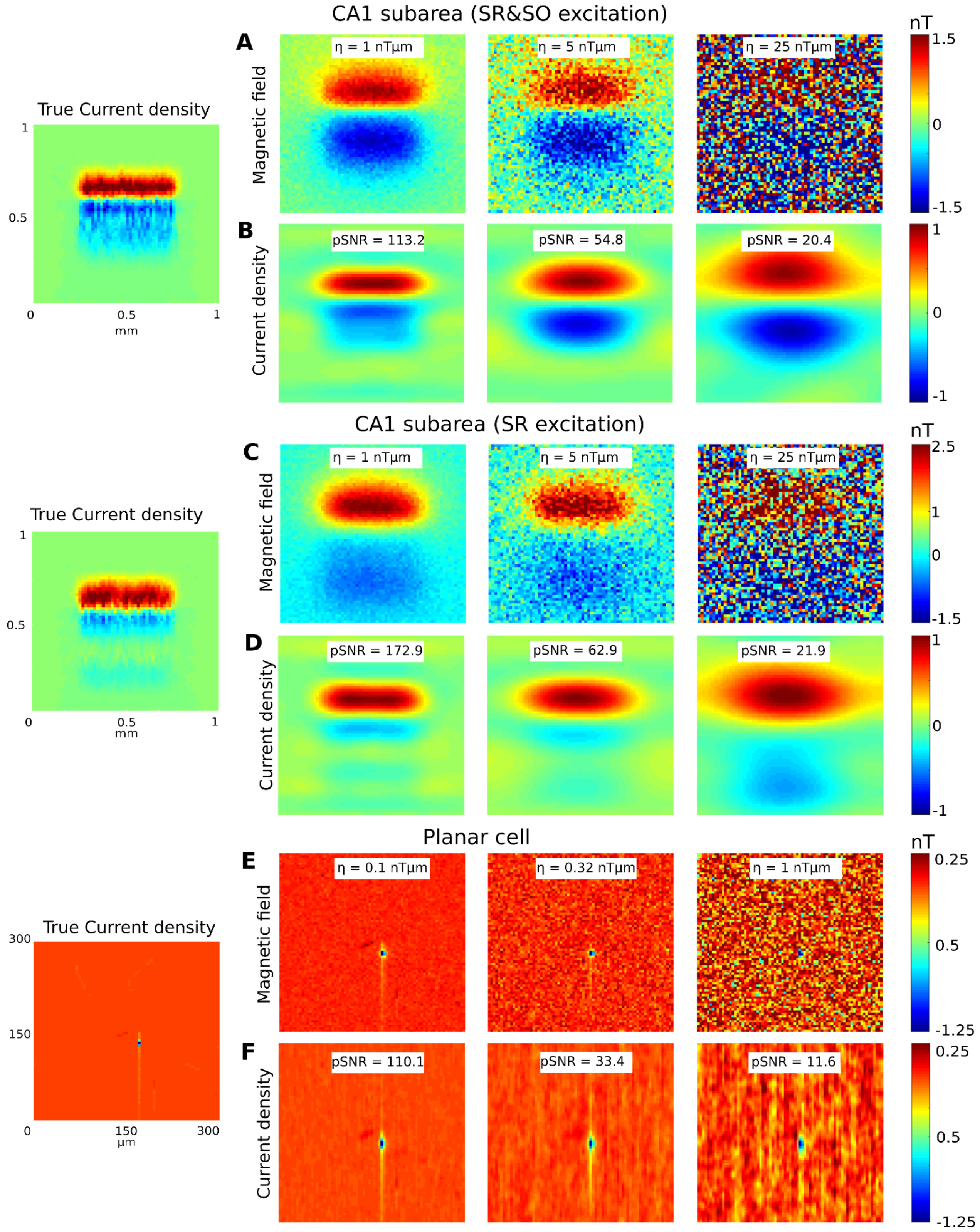

Figure S15: Reconstructed neural axial current densities at varying noise levels. **(A)** Recording from the CA1 subarea for SR&SO excitation, as mentioned in Fig.3. The true current density is determined as the total current within a voxel, divided by its volume. The in-plane resolution of the sensor is set to 15.6 μm

(64x64 pixels in 1mm^2). The B_X component of the measured magnetic field is shown for three different levels of the signal-to-noise power of the recorded magnetic field. **(B)** The normalized axial current densities reconstructed from the corresponding magnetic fields shown in A. Each plot is normalized to the maximum of its noiseless reconstruction with the same spatial filter settings. **(C)** Recording from the CA1 subarea for SR excitation, as mentioned in Fig. S13. The in-plane resolution of the sensor is set to 15.6 µm (64x64 pixels in 1mm^2). **(D)** The normalized axial current densities reconstructed from the corresponding magnetic fields shown in C. Each plot is normalized to the maximum of its noiseless reconstruction with the same spatial filter settings. **(E)** Recording of a planar cell, as simulated in Fig. 2. The cell thickness is assumed to be 2 µm. The B_X component of the measured magnetic field is shown for three different levels of the signal-to-noise power. The in-plane resolution of the sensor is set to 4 µm (75x75 pixels in 0.09mm^2). **(F)** The normalized axial current densities reconstructed from the corresponding magnetic fields in E. Each plot is normalized to the maximum of its noiseless reconstruction with the same spatial filter settings.

References

1. Hämäläinen, M., Hari, R., Ilmoniemi, R. J., Knuutila, J. & Lounasmaa, O. V. Magnetoencephalography theory, instrumentation, and applications to noninvasive studies of the working human brain. *Rev. Mod. Phys.* **65,** 413–497 (1993).
2. Ramesh M. Gulrajani. *Bioelectricity and biomagnetism*. (Wiley, 1998).
3. Clark, J. & Plonsey, R. A Mathematical Evaluation of the Core Conductor Model. *Biophys. J.* **6,** 95–112 (1966).
4. Gold, C., Henze, D. A., Koch, C. & Buzsáki, G. On the origin of the extracellular action potential waveform: A modeling study. *J. Neurophysiol.* **95,** 3113–3128 (2006).
5. Holt, G. R. A critical reexamination of some assumptions and implications of cable theory in neurobiology. (California Institute of Technology, 1998).
6. Rall, W. Electrophysiology of a Dendritic Neuron Model. *Biophys. J.* **2,** 145–167 (1962).
7. Swinney, K. R. & Wikswo, J. P. A calculation of the magnetic field of a nerve action potential. *Biophys. J.* **32,** 719–731 (1980).
8. Woosley, J. K., Roth, B. J. & Wikswo, J. P. The magnetic field of a single axon: A volume conductor model. *Math. Biosci.* **76,** 1–36 (1985).
9. Holsheimer, J. Electrical conductivity of the hippocampal CA1 layers and application to current-source-density analysis. *Exp. Brain Res.* **67,** 402–410 (1987).
10. Ness, T. V *et al.* Modelling and Analysis of Electrical Potentials Recorded in Microelectrode Arrays (MEAs). *Neuroinformatics* **13,** 403–426 (2015).

11. Nunez, P. L. & Srinivasan, R. *Electric Fields of the Brain*. *Electric Fields of the Brain: The neurophysics of EEG* (Oxford University Press, 2006). doi:10.1093/acprof:oso/9780195050387.001.0001

12. Barry, J. F. *et al.* Optical magnetic detection of single-neuron action potentials using quantum defects in diamond. *Proc. Natl. Acad. Sci. U. S. A.* **113,** 14133–14138 (2016).

13. Migliore, M., Ferrante, M. & Ascoli, G. a. Signal Propagation in Oblique Dendrites of CA1 Pyramidal Cells. *J. Neurophysiol.* **94,** 4145–4155 (2005).

14. Destexhe, A., Mainen, Z. F. & Sejnowski, T. J. in *Methods in Neural Modeling: from Ions to Networks* (ed. Koch, C. Segev, I.) 1–25 (MIT Press, 1998).

15. Spruston, N. & McBain, C. in *The hippocampus book.* 133–202 (Oxford University Press, 2006). doi:10.1093/acprof:oso/9780195100273.003.0005

16. Okada, Y. C., Wu, J. & Kyuhou, S. Genesis of MEG signals in a mammalian CNS structure. *Electroencephalogr. Clin. Neurophysiol.* **103,** 474–485 (1997).
